# Could termites be hiding a goldmine of obscure yet promising yeasts for energy crisis solutions based on aromatic wastes? A critical state-of-the-art review

**DOI:** 10.1186/s13068-022-02131-z

**Published:** 2022-04-04

**Authors:** Sameh S. Ali, Rania Al-Tohamy, Tarek M. Mohamed, Yehia A.-G. Mahmoud, Héctor A. Ruiz, Lushan Sun, Jianzhong Sun

**Affiliations:** 1 School of the Environment and Safety Engineering, Biofuels Institute, Jiangsu University, Zhenjiang, 212013 China; 2 Botany Department, Faculty of Science, Tanta University, Tanta, 31527 Egypt; 3 Biochemistry Division, Chemistry Department, Faculty of Science, Tanta University, Tanta, 31527 Egypt; 4 Biorefinery Group, Food Research Department, School of Chemistry, Autonomous University of Coahuila, 25280 Saltillo, Coahuila Mexico; 5 Institute of Textiles and Clothing, The Hong Kong Polytechnic University, Hong Kong, China

**Keywords:** Biodiesel, Circular economy, Oleaginous yeast, Insect gut symbionts, Azo dyes, Bioremediation, Lignin degradation

## Abstract

Biodiesel is a renewable fuel that can be produced from a range of organic and renewable feedstock including fresh or vegetable oils, animal fats, and oilseed plants. In recent years, the lignin-based aromatic wastes, such as various aromatic waste polymers from agriculture, or organic dye wastewater from textile industry, have attracted much attention in academia, which can be uniquely selected as a potential renewable feedstock for biodiesel product converted by yeast cell factory technology. This current investigation indicated that the highest percentage of lipid accumulation can be achieved as high as 47.25% by an oleaginous yeast strain, *Meyerozyma caribbica* SSA1654, isolated from a wood-feeding termite gut system, where its synthetic oil conversion ability can reach up to 0.08 (g/l/h) and the fatty acid composition in yeast cells represents over 95% of total fatty acids that are similar to that of vegetable oils. Clearly, the use of oleaginous yeasts, isolated from wood-feeding termites, for synthesizing lipids from aromatics is a clean, efficient, and competitive path to achieve "a sustainable development" towards biodiesel production. However, the lacking of potent oleaginous yeasts to transform lipids from various aromatics, and an unknown metabolic regulation mechanism presented in the natural oleaginous yeast cells are the fundamental challenge we have to face for a potential cell factory development. Under this scope, this review has proposed a novel concept and approach strategy in utilization of oleaginous yeasts as the cell factory to convert aromatic wastes to lipids as the substrate for biodiesel transformation. Therefore, screening robust oleaginous yeast strain(s) from wood-feeding termite gut system with a set of the desirable specific tolerance characteristics is essential. In addition, to reconstruct a desirable metabolic pathway/network to maximize the lipid transformation and accumulation rate from the aromatic wastes with the applications of various “omics” technologies or a synthetic biology approach, where the work agenda will also include to analyze the genome characteristics, to develop a new base mutation gene editing technology, as well as to clarify the influence of the insertion position of aromatic compounds and other biosynthetic pathways in the industrial chassis genome on the expressional level and genome stability. With these unique designs running with a set of the advanced biotech approaches, a novel metabolic pathway using robust oleaginous yeast developed as a cell factory concept can be potentially constructed, integrated and optimized, suggesting that the hypothesis we proposed in utilizing aromatic wastes as a feedstock towards biodiesel product is technically promising and potentially applicable in the near future.

## Background

A microbiome is defined as a microbial community that is unique to a particular habitat and is composed of yeasts, bacteria, archaea, fungi, and viruses [1]. Microbiome innovations could boost answers to recent global challenges (waste management, bioremediation, and biofuel production). For instance, screening and utilizing robust natural microbiomes to degrade or remediate aromatic pollutants may enable a more sustainable fuel industry and promote the development of a circular bioeconomy [2–6]. The use of microbiomes in environmental systems could help to reduce waste, create a zero-pollution/toxic-free environment, address the plastic challenge, and create a sustainable blue economy [7, 8]. Microbiomes, for example, can be used in environmental remediation by combining traditional techniques with microbial regeneration and incorporating microbiomes into a circular bioeconomy that valorizes waste [9, 10]. Microbiomes are also critical in the development of microbial biobased polymers that are environmentally friendly.

Aromatic compounds are important fuels and key chemical precursors for industrial organic synthesis; however, the current aromatics market is heavily reliant on fossil fuels, which will eventually contribute to global warming-related carbon emissions. On the other hand, lignin has been recognized as a drop-in substitute for conventional aromatics, with the benefits of lignin gradually becoming apparent over the last five years as a result of massive research efforts. However, the valorization of lignin, the major natural source of aromatics on earth, is being a challenge for the scientific community. Lignin is composed of aromatic compounds rather than sugar molecules (phenylpropanoids). It is a highly complex chemical compound composed of three distinct monolignol units (*p*-hydroxylphenyl [H], guaiacyl [G], and syringyl [S]) that are cross-linked. It is primarily found in secondary cell walls and makes up 10–25% of the total dry weight. Lignin is sticky and helps hemicelluloses adhere to microfibrils. Additionally, it provides structure and strength to the cell, protects it from pathogen invasion, and waterproofs the cell wall [11, 12].

Lignin-based aromatics, as a lignin-rich aromatic polymer, are the second most abundant source of carbon on the planet and provide a renewable reservoir of energy-dense substrate for green chemistry. Globally, it is estimated that 150 billion tons of lignin are produced annually [13]. The annual global production of aromatic wastes is estimated to be around fifty million tons, and this figure is expected to rise significantly in the coming years [11]. Simultaneously, lignin and its associated environmental pollutants with similar chemical structures, such as textile dye wastewater, constitute a significant aromatic pollutants on earth [14, 15]. Processing lignin is difficult due to its variability in composition and bonding structure. Furthermore, it is difficult to utilize processed lignin due to the inherent compositional heterogeneity of lignin degradation products. Currently, the majority of lignin is disposed of as waste or burned as a solid fuel in biorefinement processes. However, biological upgrading or valorization of lignin-based aromatics has advantages over conventional chemical processing or combustion in that it enables the conversion of a variety of lignin-derived substrates to high value-added products [16].

Interestingly, aromatic compound metabolism can be linked to biodiesel production [17, 18]. As a result, an emphasis has been placed on the underlying mechanisms, as well as the evaluation of the intermediates and final products produced. Biodiesel is a non-toxic, biodegradable, and renewable fuel made from vegetable oils, animal fats, and oilseed plants [19]. When burned, biodiesel emits significantly less pollution than petroleum-based diesel, whether used in its pure form or blended with petroleum diesel. It has no net effect on the amount of carbon dioxide (CO_2_) in the atmosphere and reduces the intensity of the greenhouse effect. Furthermore, biodiesel has a lower sulfur content, a higher flash point, a higher aromatic content, and is biodegradable than diesel fuel [20, 21]. However, increased biodiesel production may result in an increase in the price of edible vegetable oil. This leads to a debate about fuel vs. food.

Termites have a high capacity for biomass degradation and may thus contribute to global carbon recycling. This remarkable ability is largely attributed to the metabolic performance of their "gut digestome", which includes intestinal symbiotic microorganisms like bacterial, archaeal, yeast, or other eukaryotic symbionts, as well as the abundance of species, enzymes, and genes found in the termite's digestive tract [22, 23]. However, their highly specialized gut systems remain poorly understood in terms of their unique symbiont functions and their potential applications in biotechnology and other relevant fields, particularly for the yeast symbionts. In recent years, there has been a growing body of evidence demonstrating the importance of symbiotic yeasts, their widespread distribution in termite digestive systems, and their distinct roles in termite-symbiont interactions. Over the last two decades, additional yeast species from insect guts have been identified, as have their potential functions and relevant mechanisms of interaction with host insects and other gut symbionts. Host nutrition, essential enzymes for biomass processing, intermediate chemical compounds, suppressing external pathogens, and even colony outbreeding are examples of these functions and mechanisms [24]. Furthermore, intestinal yeasts can effectively remediate textile wastewater and decolorize both individual and mixtures of azo dyes, owing to their enzymatic activity and tolerance to high salt concentration [25]. Yeasts degrade azo dyes, which are aromatic textile wastewater pollutants by cleaving the complex compound, deconstructing the chromophores, and metabolizing the toxic aromatic intermediates produced. Finally, acetyl-CoA is synthesized as a precursor for the synthesis of lipids in the form of triacylglycerides (TAG). The degradation of azo dyes by yeast-inhabiting wood-feeding termite gut symbionts resulted in the formation of aromatic amines (AAs), which are then mineralized to CO_2_ and H_2_O [26–28]. Instead of amines, the termite gut yeasts degraded the azo dye Acid Orange 7, producing acetyl-CoA and TAG, which can be transesterified to produce biodiesel [9, 10].

The lacking of potent oleaginous yeasts to transform lipids from various aromatics, and an unknown metabolic regulation mechanism presented in the natural oleaginous yeast cells are the fundamental challenge we have to face for a potential cell factory development. One of the primary goals of the biobased economy is to develop economically viable and sustainable biotechnological processes as alternatives to oil-based chemistry. The success of this strategy will require efficient, robust, and versatile cell factories, but the limitations of conventional strain development methods will make it difficult to adapt currently used strains to such platforms. Therefore, synthetic biology is expected to enable more efficient and controllable cell factories. The yeast *Saccharomyces cerevisiae* is one of the most widely used microorganisms in biotechnology, having been successfully used to produce both bulk and fine chemicals. Yet yeast researchers face a difficult task in advancing the organism's transition from a traditional workhorse to a modern cell factory capable of meeting the requirements for next generation bioprocesses. Therefore, this review has proposed a novel concept and approach strategy in utilization of yeasts inhabiting termite gut symbionts as the cell factory to convert aromatic wastes to lipids as the substrate for biodiesel transformation.

## Energy crisis and the transition from petrodiesel to biodiesel

**Fig. 1:**
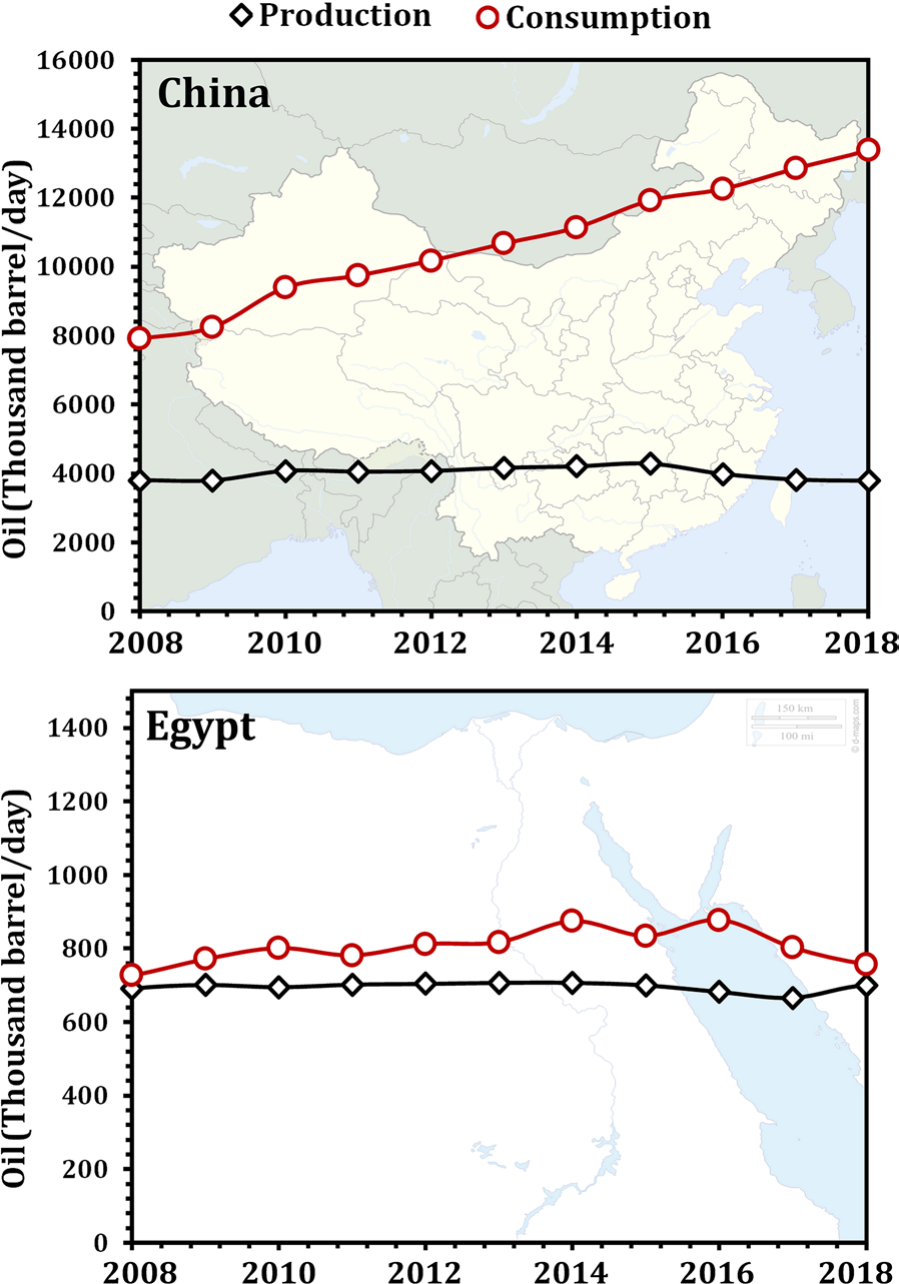
The consumption and production of oil by China and Egypt. Source of data from Refs. [30–33]

Energy is widely considered as the foundation of social development and modern human civilization, particularly in developing countries. Energy consumption is increasing at an alarming rate as a result of the rapid development of the global economy over the last few decades, resulting in a global supply and demand crisis for oil resources. The world's proven oil reserves have a life expectancy of only 53.3 years, according to oil production and consumption data, while China's reserve-to-output ratio stands at only 11.9 years [29]. China produced 3,787,000 barrels of oil per day in 2018, down 3.9% from 2008 levels (3,802,000 barrels per day). Egypt's oil production increased to 700,000 barrels per day in 2018, up 1.3% from 691,000 barrels per day in 2008. China's oil consumption (13,374,000 barrels per day) exceeded production (3,787,000 barrels per day) by 253.2% in 2018. Egypt's oil consumption, on the other hand, has increased by approximately 8.1% relative to its oil production since 2008, averaging 756.618 barrels per day in 2018, and it is expected to grow further, even after subsidies are phased out (Fig. 1) [30–33]. The world's massive energy consumption, rapidly rising oil prices, and growing concern about dwindling fossil fuel reserves have redirected global attention toward the search for alternative energy sources. As a result, developing renewable technologies to replace current technologies is a critical challenge for the twenty-first century.

**Fig. 2:**
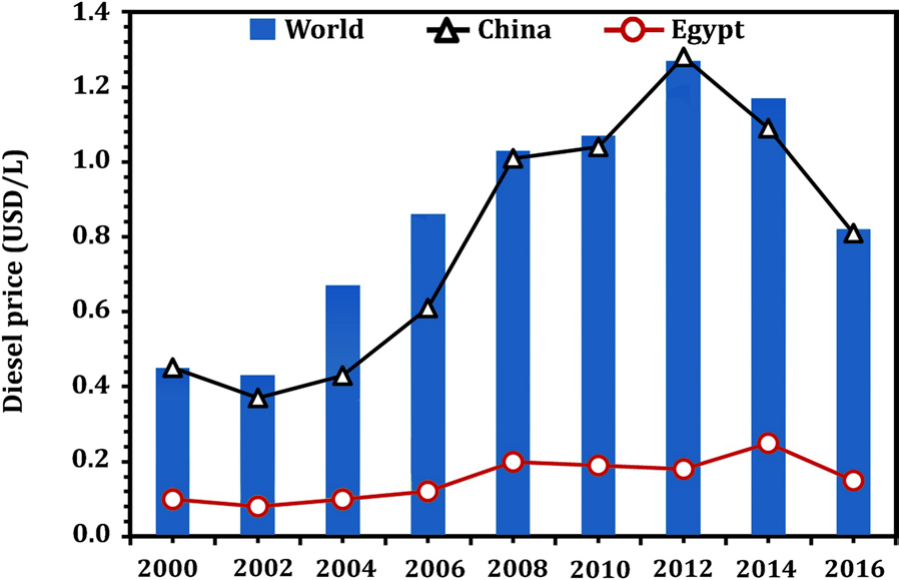
The global diesel price in comparison to China and Egypt. Source of data from Refs. [35–38]

Petrodiesel has been shown to emit a significant amount of hazardous pollutants, including carbon monoxide (CO), hydrocarbons (HC), particulate matter (PM), sulphur dioxide (SO_2_), nitrogen oxides (NOx), and greenhouse gases (GHG). All of these pollutants have been shown to be harmful to the environment and human health. Concerning the environment, petrodiesel combustion can also result in the emission of CO_2_. In 2020, the global CO_2_ concentration reached a record high of 413 parts per million, with CO_2_ emissions from transportation, including the combustion of fossil fuels, accounting for the second largest source of global CO_2_ emissions [34]. The global diesel price, on the other hand, varied from country to country. In Fig. 2, the global diesel price (2000–2006) in comparison to prices in China and Egypt is given [35–38]. In China, the diesel price was slightly higher and fluctuated less than the global price, peaking at 1.28 USD/L in 2012. Diesel prices, on the other hand, were significantly lower in Egypt, where the highest price was recorded in 2014 (0.25 USD/L), most likely due to governmental support, putting a strain on the Egyptian economy.

**Fig. 3:**
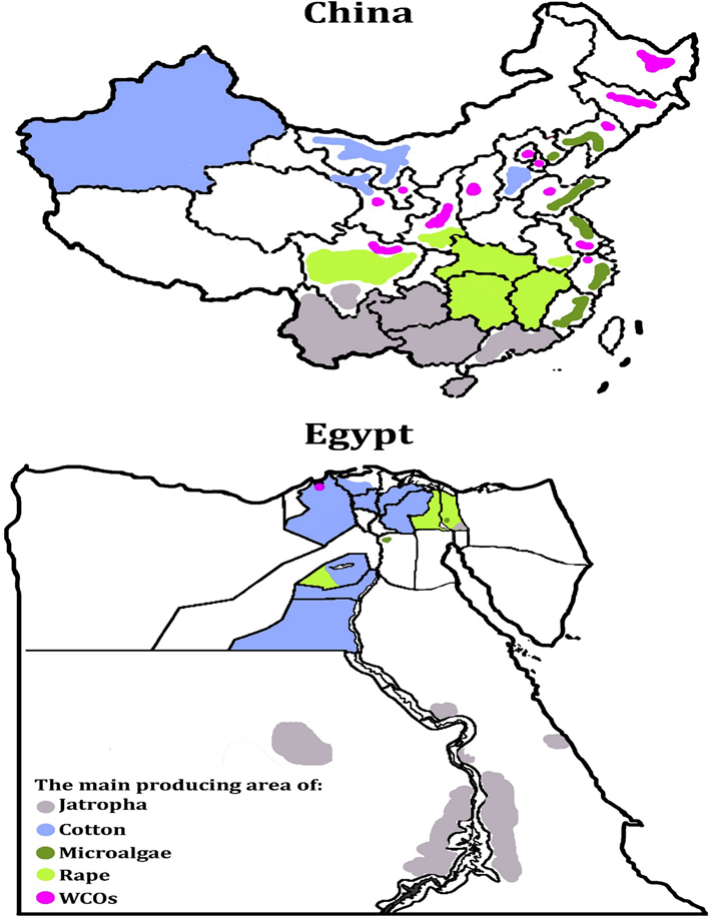
The regional distribution of raw materials that could be used in the production of biodiesel in Egypt (**A**) and China (**B**). Source of data from Refs. [43–50]

Biodiesel, on the other hand, is being introduced to the public due to its benefits, which include low pollutant and greenhouse gas emissions, as well as its renewability. Table 1 summarizes the advantages and disadvantages of petroleum diesel and biodiesel [39, 40]. When compared to petrodiesel, pollutants such as CO, HC, PM, and SO_2_ can be reduced by up to 40% on average (SO_2_ can be reduced by up to 100%) [41]. Furthermore, when compared to petroleum diesel, biodiesel has the potential to reduce CO_2_ emissions by 78.45% [34]. Because it could be made from vegetable oil, animal fats, or any other substance containing long chain fatty acids, biodiesel was considered a renewable resource [42]. Figure 3 depicts the regional distribution of raw materials used in the production of biodiesel [43–50]. In Egypt, biodiesel can be made from a variety of feedstocks, including cotton, Jatropha, rapeseed, microalgae, and waste cooking oils (WOCs). Upper and lower Egypt could practice jatropha, cotton, and rapeseed agriculture. Microalgae and WCOs, on the other hand, are considered uncommon in Egypt (Fig. 3A) [43–46]. When agricultural characteristics and regional diversity are considered, it is difficult to focus on a single type of biodiesel feedstock in China. Western China, as shown in Fig. 3B [47–50], is a cotton-producing region, whereas the Yangtze River Basin is a rapeseed-producing region. Simultaneously, Jatropha is abundant in Southwest China, implying that using the appropriate feedstock in a reasonable manner can result in economic benefits for biodiesel production.

**Table 1:** The benefits and drawbacks of petrodiesel and biodiesel

Fuels	Advantages	Disadvantages
Petrodiesel	Low feedstock cost	High emissions of GHG and pollutants
	Low viscosity	High toxicity
		Low biodegradation rate
		Non-renewable fuel
		Low flash point
Biodiesel	Low emissions of GHG and pollutants	Expensive feedstocks (first- and second-generation biodiesels)
	Low toxicity	Requires land for feedstock cultivation (first- and second-generation biodiesels
	High biodegradation rate	Some feedstocks have low crop yields (first- and second-generation biodiesels)
	Renewable fuel	
	High flash point	
	Can be used in diesel engine

**Fig. 4:**
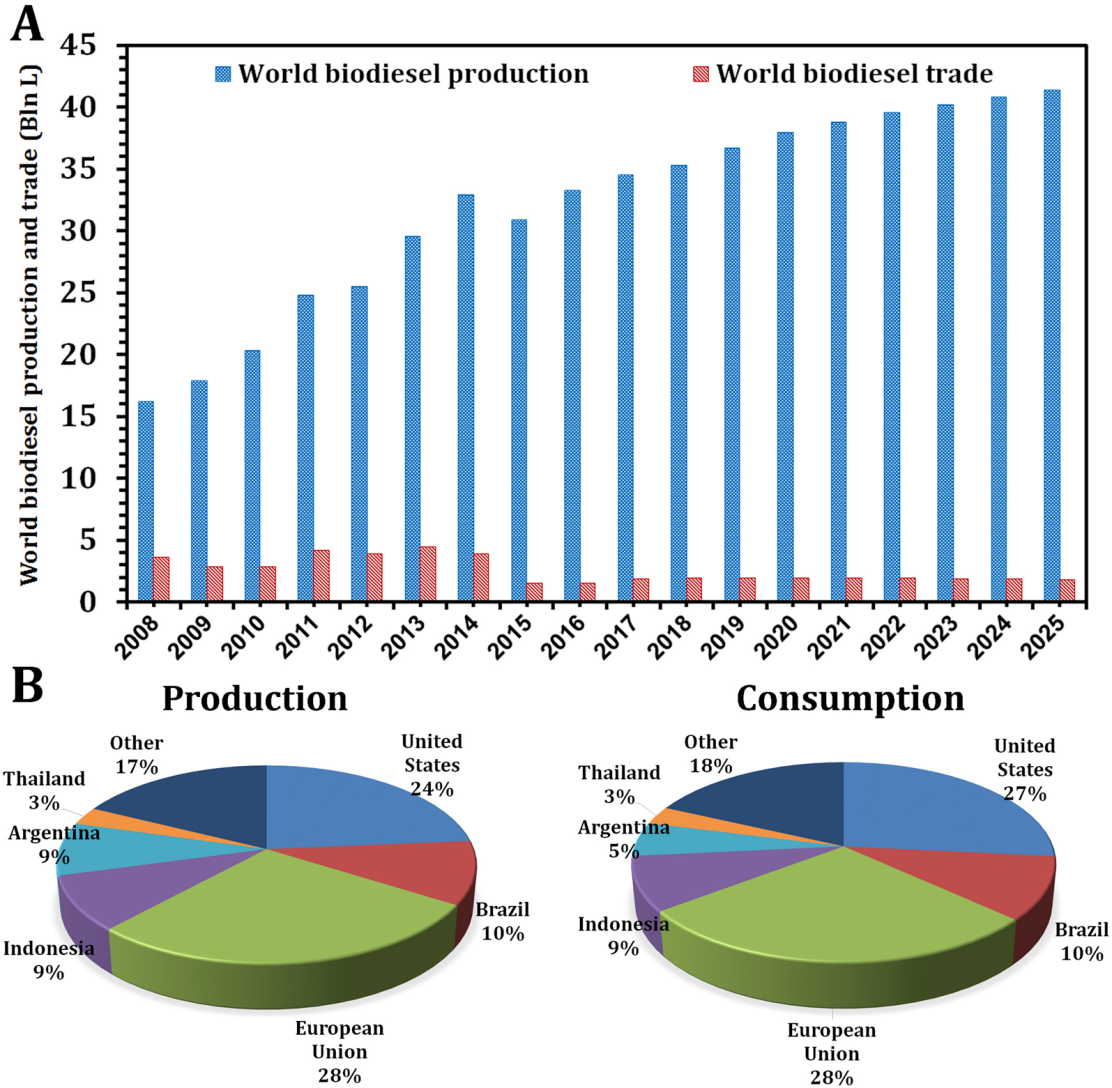
Production and trade of biodiesel on a global scale (**A**). Regional distributions of global biodiesel production and consumption in 2025 (**B**). Source of data from Ref. [52]

**Fig. 5:**
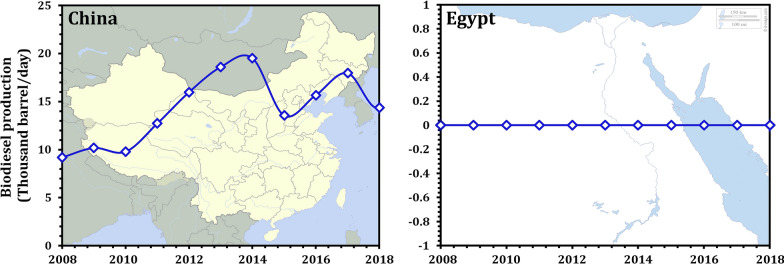
Biodiesel production in China and Egypt (2008–2018). Source of data from refs. [53, 54]

Global biodiesel production is increasing rapidly to meet demand, having increased from 15,000 barrels per day in 2000 to 289,000 barrels per day in 2008 [51]. In 2020, the world's biodiesel production is expected to reach 37.9 billion liters [52]. However, it is expected to reach 41.4 Bln L by 2025 (Fig. 4A). Additionally, by 2025, the European Union and the United States of America are expected to be the largest producers and consumers of biodiesel (Fig. 4B). China and Egypt, on the other hand, require additional efforts to improve their biodiesel production. Since 2008 to 2018, China's biodiesel production has fluctuated. In 2008, production was 9200 b/d; by 2018, it had increased to 14,370 b/d, a 56.2% increase. However, between 2000 and 2019, Egypt’s biodiesel production remained stable at approximately 0 b/d (Fig. 5) [53, 54].

Biofuels, including biodiesel, have traditionally been classified into three generations based on their feedstock and manufacturing technologies. Biodiesel produced in the "first generation" can be made from edible feedstocks such as palm oil, rapeseed, soybean, and corn. However, using edible oil crops to produce biodiesel has raised several controversial issues, including GHG emissions, land use change (LUC), food competition/price inflation, high feedstock/biodiesel costs, and water consumption [55]. The development of "second-generation" biodiesel has been accelerated due to the rising cost of edible plant oils, as well as public debate over the "food vs. fuel". Biodiesel of the second generation can be made from non-edible feedstocks such as jatropha, neem, rubber seed, karanja, and waste oils such as cooking grease and animal fats. These oils, however, may not be abundant enough to meet global demand, and animal fats perform poorly in cold weather. Additionally, the use of non-edible plant oils in the production of biodiesel has raised several controversial issues, including the GHG footprint, LUC, and fertilizers/pesticides [56].

## Oleaginous yeasts as an alternative to algae for biodiesel production

Third generation biodiesel can be produced using feedstock that does not compete with crops for land. It is being developed by bacteria, algae, yeasts and other fungi that accumulate oil. Single cell oils (SCO) are microbial systems that produce and store oil [55]. SCO have received a great deal of attention in recent years, largely as a result of the increasing price of petroleum. Cost estimation for SCO has proven challenging, with biodiesel cost projections ranging from $1 to $80 per gallon [57]. In China, the cost of producing SCO from lignocellulosic biomass is 7500 RMB (US$ 1230) per ton, which includes the cost of biomass procurement and processing [58]. Oleaginous microbes are capable of converting CO_2_, sugars, and organic acids to SCO. While some species synthesize neutral lipids intracellularly on a continuous basis, the majority of cell types require stressors such as nutrient deprivation to stimulate lipid synthesis [59]. Once the cells have produced the lipid, they are harvested and lysed with a solvent, mechanically, enzymatically, or by another method, which releases the lipid. After separating the lipid from the cell fraction, the neutral lipid is chemically refined to form an ester or other desired molecule by separating the glycerol from the individual fatty acids [60]. While the methodology has been established, the current barrier to commercializing SCO is developing a cost-effective system comparable to petrodiesel. This can be accomplished by developing strains capable of converting low-cost substrates, rapidly growing to high density, and producing increased amounts of neutral lipid, as well as by developing improved harvesting and dewatering technologies [61, 62].

Photoautotrophic, heterotrophic, and mixotrophic cultivation methods are used to cultivate algae. Outdoor racetrack ponds, enclosed photobioreactors, and enclosed dark fermentors are all viable cultivation techniques. However, there are a number of restrictions that must be considered when selecting an algal biodiesel production method, as listed below:

- Microalgae cultivation requires a large raceway pond capable of producing a significant amount of algal biomass for biodiesel production [63].
- Only a few microalgae contain a high concentration of fatty acids or lipids. As a result, the quantity produced will be reduced, raising the cost of biodiesel production [64].
- The process of converting lipids to biodiesel entails a number of steps and a number of different chemicals [65].
- It is also necessary to purify biodiesel before it can be used in vehicles to ensure that it is pure [66].
- Biodiesel's energy value is also required for quality control and production. This will necessitate the completion of a time and expense analysis [67].
- In each batch of microalgae cultivation, the amount and quality of lipids produced vary depending on climatic conditions and the generation of algae used [68].
- Vehicle engines must currently be replaced with engines that are compatible with biodiesel. Such a replacement, on the other hand, is a time-consuming process.
- Algae processing for biodiesel is a time-consuming process that must be avoided due to microbial contamination [69].
- As the cost of manpower and materials for pond and photobioreactor construction rises, it is possible that production costs will increase. Depending on the environmental conditions, this will raise the cost of production each year.
- In some countries, research on genetically modified microalgae is prohibited in order to preserve indigenous wild species that produce less lipid [70].
- With regard to cultivation, numerous obstacles must be overcome and untangled in order to develop an effective mass cultivation technology for microalgae. This requires a comprehensive understanding of photosynthetic, structural, and functional characteristics of microalgae, as well as the target strain's dominance over alien strain/bacteria contamination and, of course, biomass productivity per unit area.
- Macroalgae cultivation varies by season and country, depending on the sea environment. Based on their native species, only a few macroalgae species are cultivable worldwide [71].

**Fig. 6:**
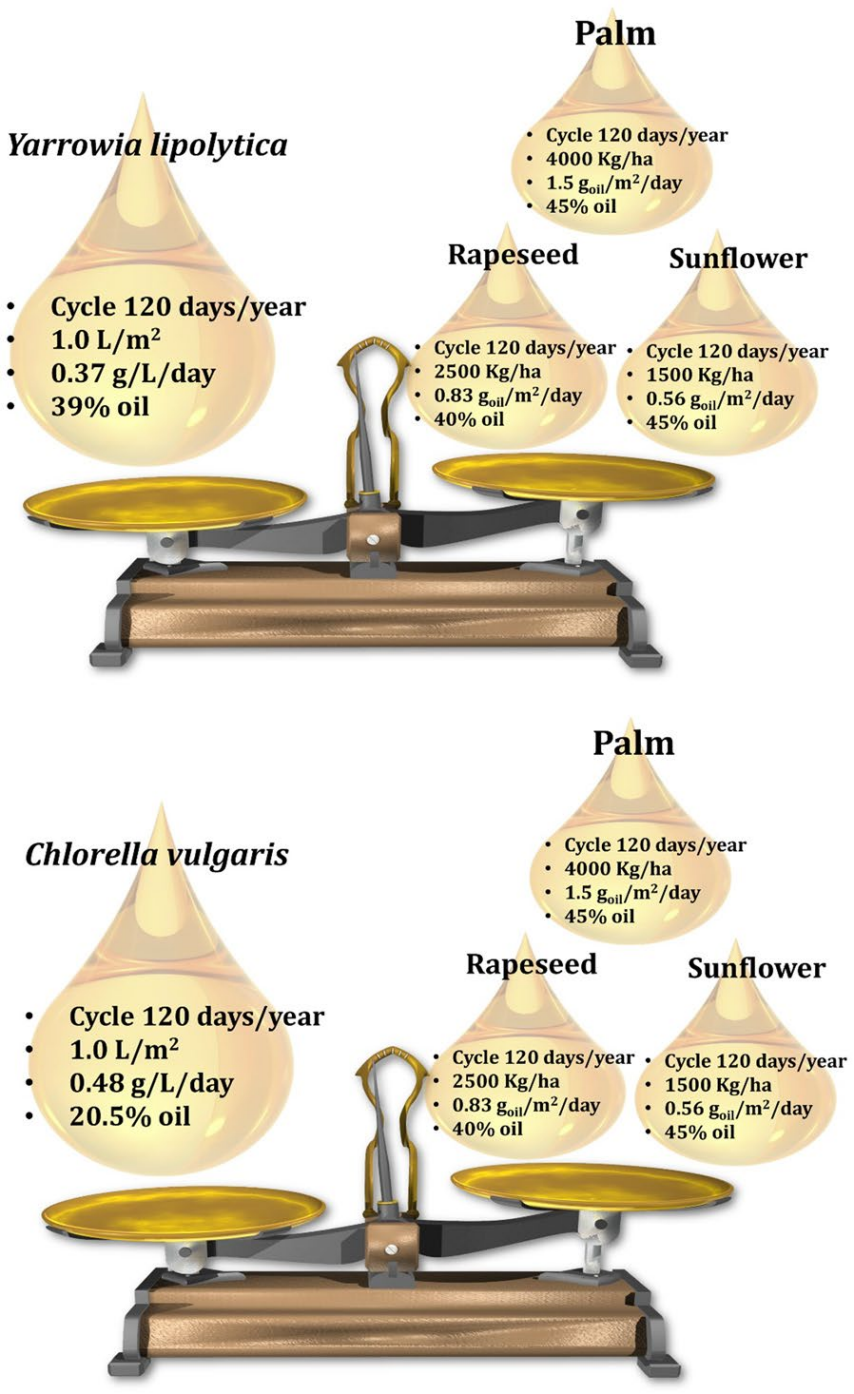
Oleaginous yeast lipid content and productivity compared to oleaginous microalgae and conventional feedstocks. Source of data from Refs. [78, 79]

In order to better understand third-generation biodiesel, oil-accumulating yeasts are being used in the research. Oleaginous yeasts are capable of converting CO_2_, sugars, and organic acids into oil. The ability to convert carbohydrates to lipids, as a result, makes them advantageous for the production of biofuels [42, 72]. Certain yeast species can accumulate a wide variety of lipids between 20 and 80% (w/w) of their dry weight [73]. Despite the fact that the process is similar in most species, the mechanism by which these microorganisms accumulate lipids is not well understood. Oleaginous yeasts are considered potential oil exploration organisms due to their rapid growth rate, ability to grow on a variety of different substrates, and high cell density [2, 74, 75]. Scaling-up this process is easier with yeasts than with microalgae, as internal contamination is less common and is easier to control at low pH [76, 77]. The comparison of the lipid productivity of different emergent matrices to a conventional feedstock is shown in Fig. 6 [78, 79]. The lipid content and productivity of oleaginous yeasts such as *Yarrowia lipolytica* (Fig. 6A) were compared to the lipid content and productivity of oleaginous microalgae such as *Chlorella vulgaris* (Fig. 6B). The production of biodiesel from microbial oil is fraught with technical difficulties, owing primarily to the high cost of fermentation. As a result, less expensive feedstock such as agricultural residues and other aromatic wastes must be used as a substitute for expensive feedstock. There are several advantages to consider when selecting an oleaginous yeast for biodiesel production, which are summarized below.

- It is possible to genetically improve the majority of yeast species, making them suitable for large-scale fermentation and genetic improvement [80].
- Oleaginous yeast species produce lipids that are chemically and energetically similar to animal/plant oils [81].
- It is possible to use oleaginous yeasts in place of a variety of vegetable oil-based applications without endangering food resources [82].
- In the case of oleaginous yeasts, when grown on low-cost wastes, they can be ideal bioeconomic feedstocks, contributing significantly to waste pollution management [81].
- Some oleaginous yeast species have gained great attention in recent years, and some, such as *Yarrowia lipolytica*, *Lipomyces starkeyi*, and *Rhodotorula toruloides*, are now being used on an industrial scale for biodiesel production [83]. These species have benefited from features such as ample genetic resources and molecular responses to a variety of growth conditions. Such characteristics have aided in the development of tailor-made gene-editing technologies.

In both bacterial and eukaryotic metabolism, the biosynthesis of long-chain fatty acids from acetate, followed by esterification to produce TAG, is an important step in the energy production process. The majority of organisms produce significant amounts of TAG, but only in the quantities required for cell membrane synthesis. However, the majority of oleaginous yeast species, such as *Yarrowia lipolytica* and *Cryptococcus curvatus*, are capable of producing excess TAG and storing them intracellularly in lipid vesicles [22]. These lipids can be used as a feedstock for the production of industrial biodiesel via conventional transesterification techniques. *Cryptococcus curvatus* is particularly interesting based on the ability of this oleaginous yeast species in utilizing effluent from an anaerobic digestion process that accumulates volatile fatty acids (VFAs) as a growth substrate for yeast, allowing the yeast to produce biodiesel [84].

Oleaginous yeasts have the ability to produce a large amount of lipid with the desired composition for biodiesel production, as well as the ability to efficiently metabolize low-cost feedstock [85]. Additional advancements, primarily in the areas of cost reduction and increased productivity, are expected to continue until industrialization is completed. Furthermore, many yeast species are underexplored, so new sources of promising isolates should be thoroughly investigated. Yeasts derived from termite gut symbionts are one such extraordinary example.

## Yeast–termite association and significance

Termites rely on mutualistic intestinal microbes for nitrogen and metabolism [86]. Termites (Isoptera) are traditionally classified as "lower" or "higher" termites based on the symbionts they coexist with [87]. Both lower and higher termites are capable of harboring prokaryotes in their guts, whereas flagellate protists are absent from the guts of higher termites, which rely heavily on gut bacteria for plant degradation [88]. It is reasonable to assume that the gut microbiota of wood-feeding higher termites would be the most lignocellulolytic in nature, as demonstrated in the *Nasutitermitidae* species' gut microbiota [89]. Structural changes in the side-chains of lignin have also been observed in a number of termite species. Furthermore, a large number of isolates obtained from a variety of wood-feeding termite gut symbionts were shown to be capable of mineralizing lignin-derived monoaromatic and dimeric model compounds in a short period of time [22].

**Fig. 7:**
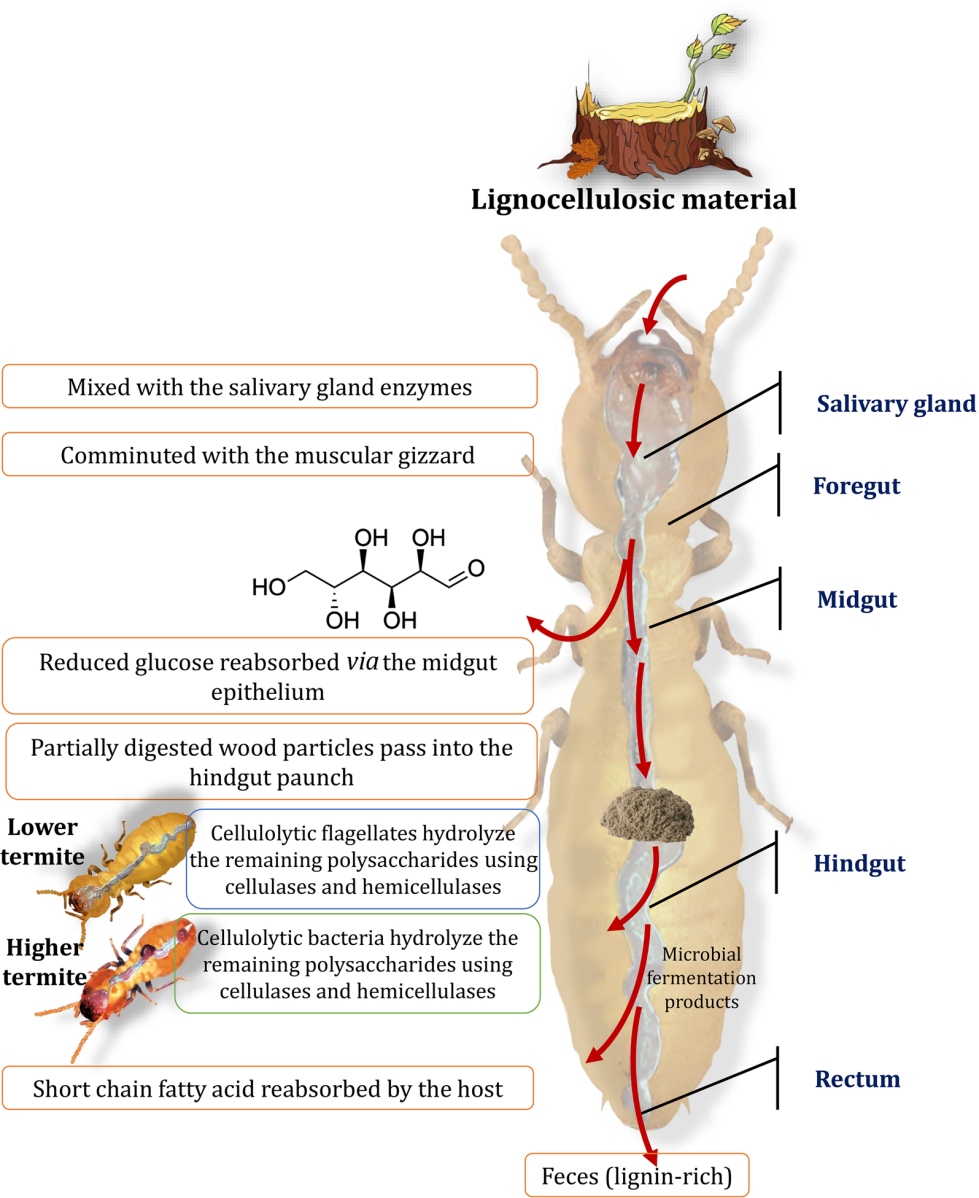
Different lignocellulosic biomass digestion strategies in termites involve both the host and its gut microbiota

There is a scarcity of information on yeasts (single-cell fungi) in termite guts. Nonetheless, preliminary research has revealed that yeast isolates are found in large numbers in both lower and higher termites in various systematic positions. Yeasts are most likely members of the hindgut native symbiotic microbiota, since they have been isolated on a regular basis [90]. Among the microorganisms available, yeasts are in high demand for a variety of biotechnological applications [91]. They are common in detritus or wood-feeding insects' guts, and they probably have a crucial role in the digestion process through their lignocellulose-degrading activities despite the fact that none of these yeasts produce all of the hemicellulose-degrading enzymes [4, 92]. Therefore, due to the synergistic activities with other gut symbionts, glycolytic enzymes can degrade these substances. For example, *Sporothrix albicans* and *Debaryomyces hansenii, Rhodosporidium* spp*.*, *Trichosporon* spp. could also be found in the hindgut of the termites from the families of *Mastotermitidae*, *Rhinotermitidae*, and *Kalotermitidae* [93]. Therefore, the termite intestines can be seen as a sophisticated ecosystem with complex microbial communities. During the passage through the digestive tract of food, both lower and higher termites degrade cellulose and hemicellulose, but with different digestive strategies (Fig. 7).

The termite symbiotic system offers numerous opportunities for the discovery of new genes and enzymes. Recent advances in -omics, such as metaproteomics, metagenomics, and metatranscriptomics, have been used to elucidate the nature of this system [94]. Characterization and genetic engineering of these enzymes are essential for improving their suitability for industrial use. The synergistic collaboration of enzymes in lignocellulose digestion should also be considered to maximize the efficiency of biomass utilization. While the association of some termites with fungi is well established, there are only a few reports of yeast–termite associations. Nowadays, eukaryotic expression systems, particularly yeasts inhabiting wood-feeding termite guts, are gaining increased attention for the enzymes they produce that have significant industrial applications, including biofuels and bioremediation [10, 28].

**Fig. 8:**
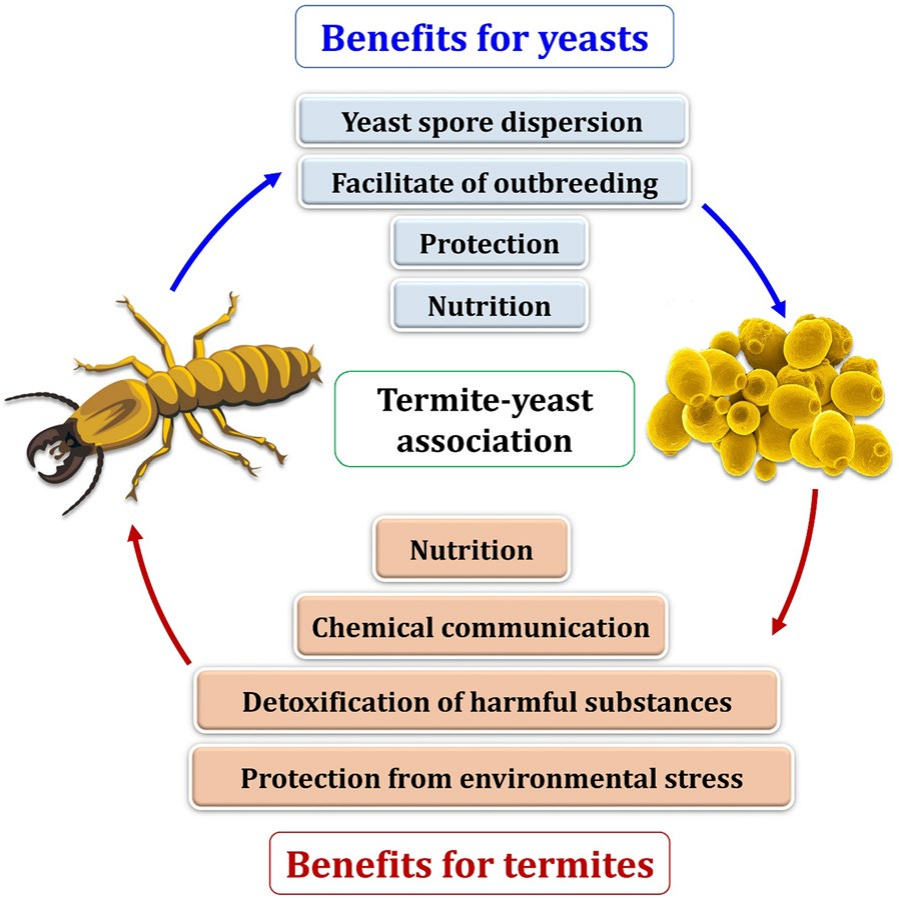
Interactions between termites and yeast

Figure 8 depicts the exceptional benefits of yeast symbionts provide to their termite hosts. Yeast symbionts can provide nitrogen and vitamins to their termite hosts, as well as degrade cellobiose and produce a variety of enzymes such as lipase, phosphatase, glucosidase, and trypsin that aid the host in digestion and detoxification of a variety of compounds [24, 90]. Due to the inability of termites to degrade lignin, ligninases-producing yeasts in wood-feeding termite guts are essential candidates for assisting such hosts in entering the plant, providing an explanation for entry in the absence of suitable enzymes [22, 95]. Yeasts in wood-feeding termite gut symbionts may also aid in the digestion of difficult substances such as wood components (e.g., cellulose, hemicellulose and xylans) [96, 97]. Despite the fact that termites are capable of producing their own cellulase, no endogenous hemicellulases have been discovered [98]. Thus, yeasts found in termite guts can provide the enzymes required for hemicellulose digestion. Yeasts, in particular, have been shown to contribute to the degradation of these wood components [99]. The ability of several yeast species isolated from termite guts to digest lignin-based aromatics, xylan, and hemicellulose was determined [28, 100].

Despite extensive research, little is known about the extent to which yeasts benefit from termite associations (Fig. 8). Termites can vector yeasts and potentially protect them from unfavorable environments by living inside their guts. Furthermore, the termite gut may provide a favorable environment for yeast growth and survival, providing the organism with a consistent source of nutrition [101]. Termite guts may provide a unique environment for yeasts that cannot survive in other environments. New yeast species were discovered among termite gut symbionts [90, 96, 97]. Termites may also affect yeast biodiversity, altering the density and composition of yeast populations. The association between termites and yeasts does not appear to be coincidental. There are three hypotheses for endosymbiotic yeast–termite associations. Yeast symbionts were thought to be descended from termite commensals or pathogenic parasites, as well as phytopathogenic or saprophytic fungi. The association may also arose as a result of termite feeding habits [22]. Given what we know about these associations, it is not surprising that adaptation mechanisms are still a mystery.

## Biodiesel production advances based on toxic aromatic compounds

**Fig. 9:**
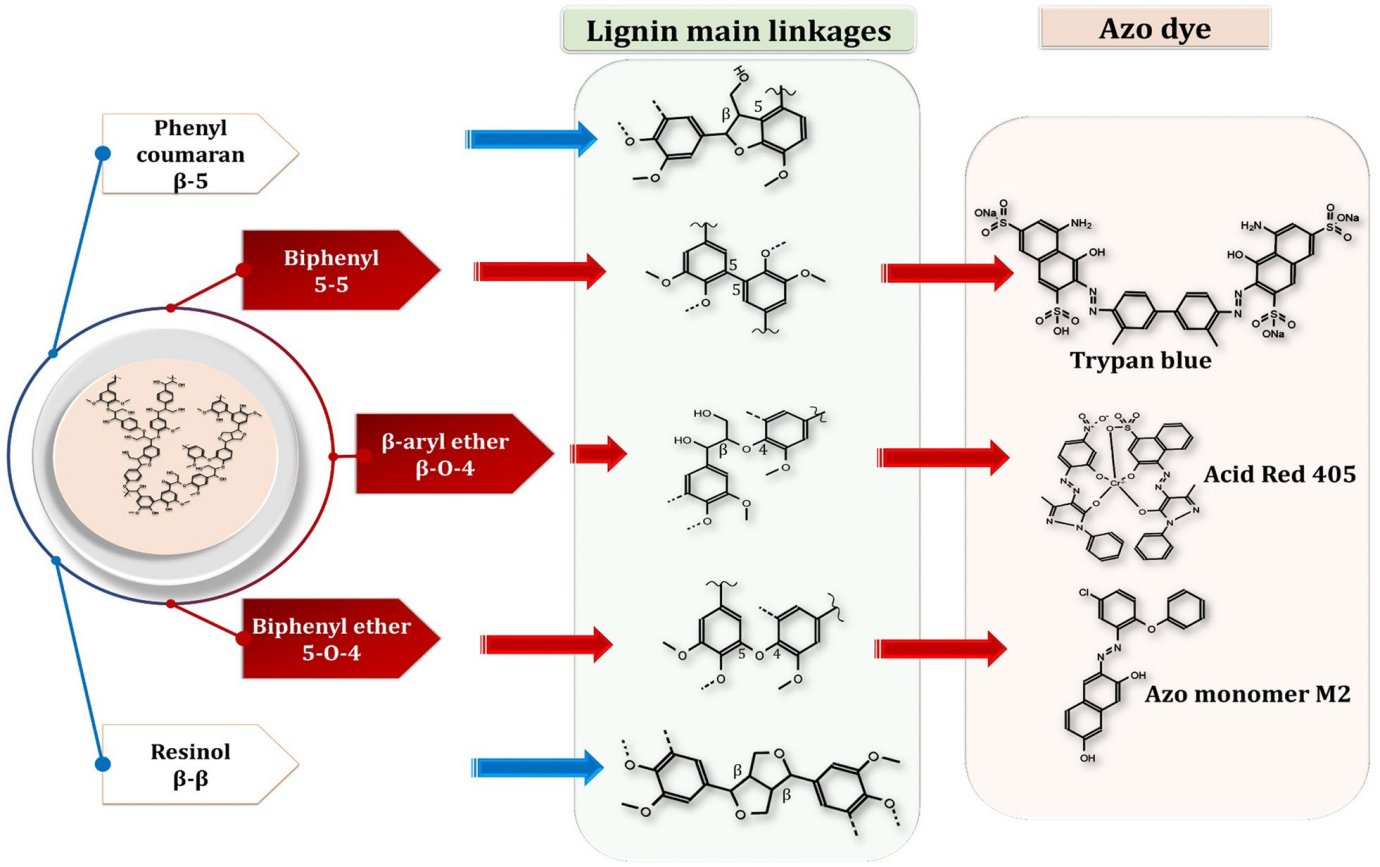
The aromatic chemical structure of lignin and azo dye

Contemporary science is increasingly focusing on not only the utilization of industrial and human wastes, but also on the valorization of aromatic pollutants such as lignin derived from lignocellulosic biomass and textile azo dyes for lipid production by oleaginous microorganisms as a substitute for fossil fuels. Lignin, a naturally occurring polyphenolic compound found in lignocellulosic biomass, and lignin-derived compounds are also significant sources of aromatic compounds that are frequently recalcitrant to biological degradation. Azo dyes, on the other hand, are a class of compounds distinguished by the presence of azo bonds (–N=N–) in association with aromatic groups, which renders them resistant to the majority of biological and chemical treatments. Figure 9 illustrates the similarity in the aromatic chemical structure of lignin and azo dye.

## Lignin as aromatic polymer derived from lignocellulosic biomass

Anthropogenic aromatic pollutants have serious environmental and human health consequences. These pollutants can enter soil and water habitats from a variety of sources, including textile wastewater, oil spills, industrial waste, and pipeline leaks. There are two categories of aromatic compounds based on the arrangement of benzene ring: monoaromatics, which contain a single aromatic ring, and polycyclic aromatics, which contain two or more aromatic rings [102]. On the other hand, heterocyclic aromatic compounds are monoaromatic or polycyclic aromatic compounds that contain atoms other than carbon, such as nitrogen or sulphur [103]. Polycyclic aromatic hydrocarbons (PAHs) are a large class of compounds with multiple fused benzene rings that are produced by a variety of natural and anthropogenic activities [104]. While volcanic eruptions and forest fires are natural sources of PAHs, the primary anthropogenic sources are biomass burning, coal combustion, petroleum volatilization, and vehicle exhaust.

**Fig. 10:**
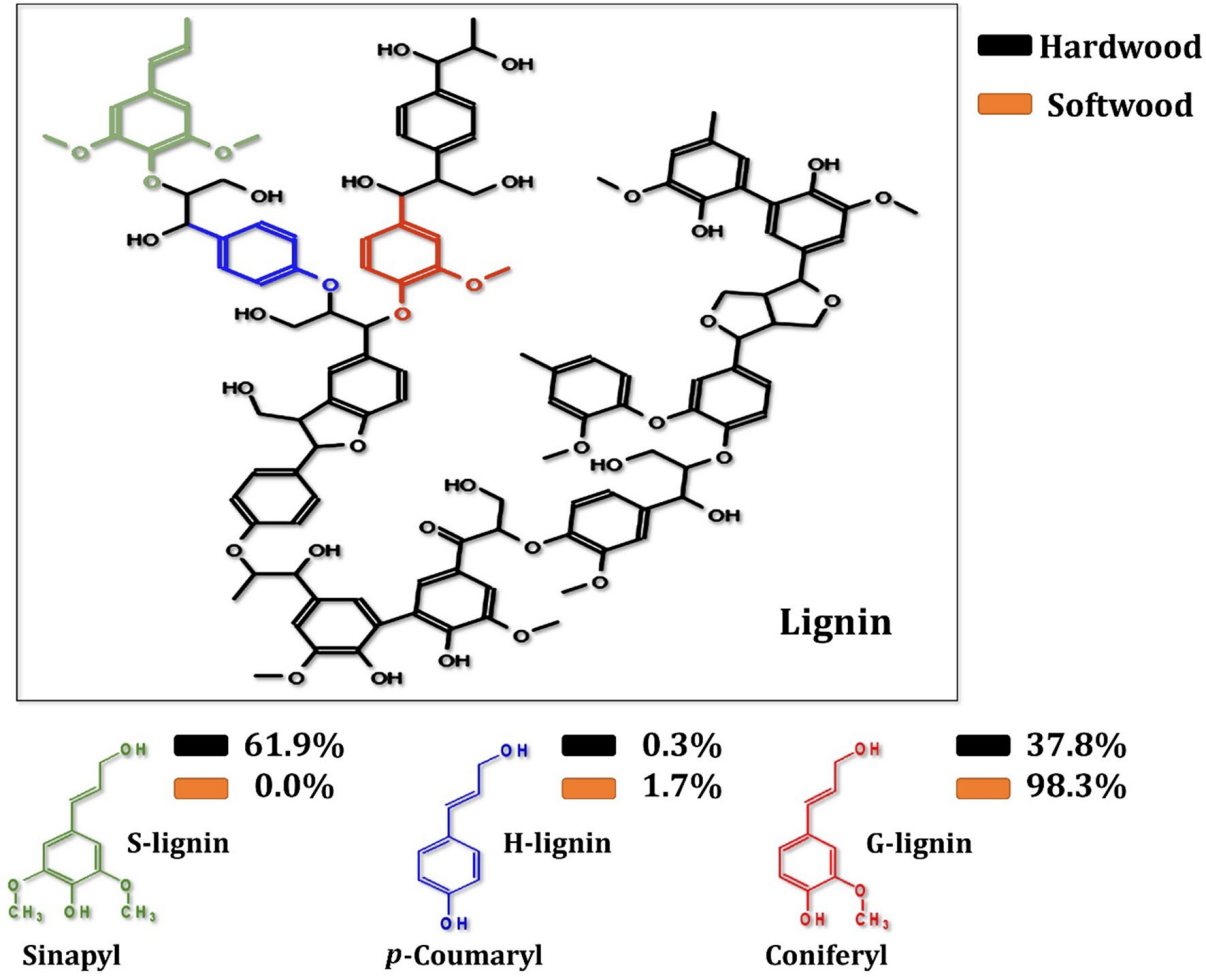
The fundamental structure and compositions of lignin units in various species

Lignocellulosic biomass is a renewable energy source that is primarily composed of cellulose, hemicelluloses, and lignin. Lignin is an aromatic polymer that contributes rigidity, water transportation, and resistance to microbial invasion. Lignin is the most abundant renewable biopolymer of aromatic compounds on earth, accounting for up to 32% of the dry weight of softwoods [105]. Aromatic compounds are also found in trace amounts as side groups in plant biomass, such as xylan, tannins, and pectin [106]. Although lignin units are simple phenylpropane molecules, the polymer produced is extremely complex in three dimensions, and only extracellular enzymes can access the polymer surface. The lignin bonds also limit enzymatic activity, leading to lignocellulosic waste accumulation and environmental burden [107]. Lignin is a highly branched aromatic polymer that is composed of three main monomers, namely guaiacyl (G), syringyl (S), and p-hydroxyphenyl (H), which are linked through a variety of linkages that differ among plant species (Fig. 10). Filamentous fungi and yeast are the most efficient lignin degraders [108]. The carotenogenic yeast *Rhodosporidium toruloides* showed the ability to degrade lignin-based aromatics into high value-added products used in the production of biofuels [109]. Because aromatics derived from lignocellulosic biomass have a wide range of industrial applications, aromatic polymers have significant potential for the sustainable production of valuable compounds in bioenergy.

By 2025, the World Bank estimates that waste generation will exceed 2.6 billion tons due to population growth [110]. Around 60% of all waste generated on a global scale is lignin-based aromatics (organic waste from agriculture and textile industry) [111]. Currently, these organic residues are composted or incinerated, resulting in secondary wastes or methane and CO_2_ emissions [112]. As a result, recycling lignin-based aromatic wastes into high value-added products is necessary to minimize environment pollutants and conserve energy. In addition, one of the primary obstacles to economically viable SCO is the search for suitable low-cost carbon sources for oleaginous fermentation via various fermentation pathways. The use of lignin-based aromatic wastes as a feedstock can significantly increase industrial-scale SCO production. However, a number of challenges must be overcome before biodiesel-based aromatic compounds become a reality in the near future.

**Fig. 11:**
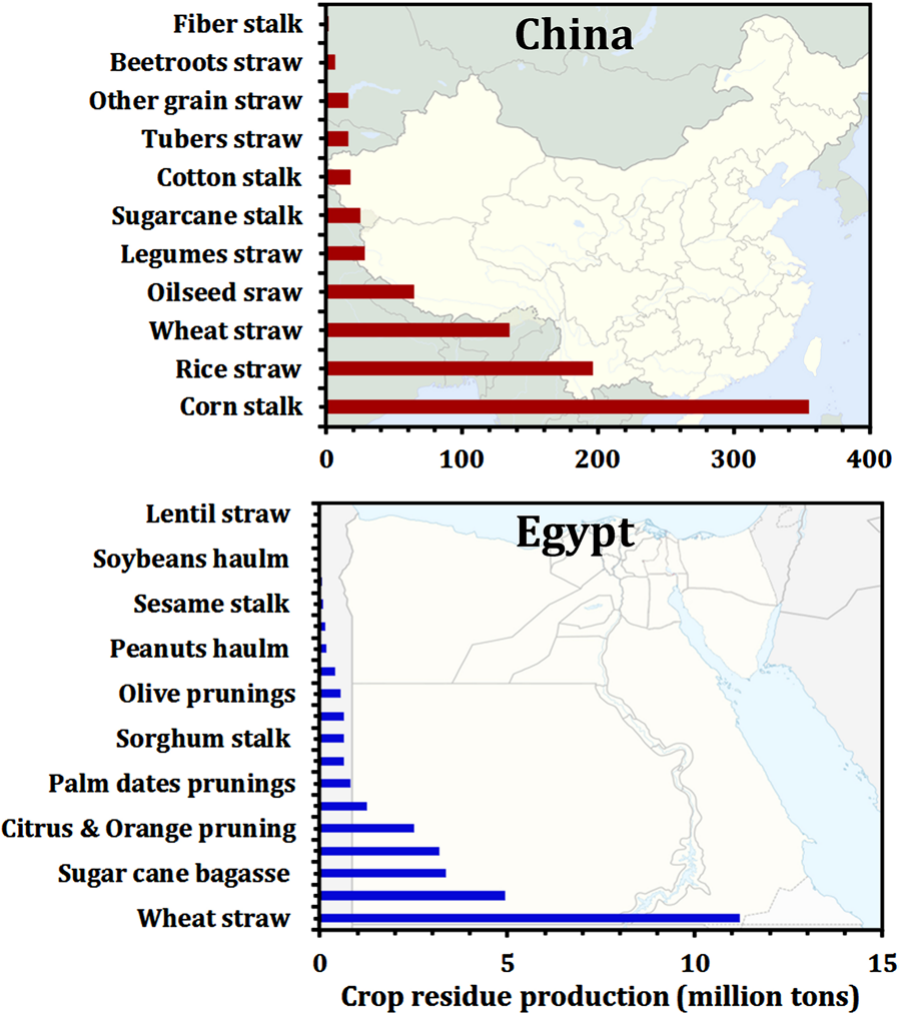
The availability of lignocellulosic biomass as a feedstock for biodiesel in China and Egypt. Source of data from Refs. [113, 114]

Lignocellulosic biomass is a plentiful source of renewable energy. Once released from carbohydrate polymers that are tightly bound to lignin, monomer sugars can be used as a carbon source for SCO synthesis. Corn stalk accounted for the largest proportion of agricultural crop residues in China, accounting for over 354.49 million tons, or 41.66% of total theoretical quantities, followed by rice straw at over 195.76 million tons (23.01%) and wheat straw at over 134.76 million tons (15.84%); cotton stalk accounted for 17.35 million tons (2.04%); oilseeds crops straw (primarily canola and peanut) accounted for 64.60 million tons (7.59%); and legumes straw (1.83%) as shown in Fig. 11 [113]. However, in Egypt, wheat straw and maize stalk weighing 11.2 and 4.9 million tons, respectively, representing 36.7 and 16.2% of total lignocellulosic residue, are depicted in Fig. 11, along with the remainder of lignocellulosic residue [114]. Enzymatic catalysts are typically used to liberate sugars from lignocellulose. Cellulolytic and hemicellulolytic enzymes, on the other hand, are frequently inefficient on their own. Thus, pretreatment is essential to enhance the biomass digestibility are sugar liberation [115].

Degradation products such as furans, carboxylic acids, or phenols produced by certain pretreatment procedures can have a significant impact on fermenting microorganisms. Numerous approaches, such as detoxification and adaptation, are being investigated in this context to alleviate the inhibitory effect of these products on microorganisms when lignin-based aromatics are employed as the raw material. It appears that despite the inhibitors, lipids can be produced from lignocellulosic waste, and several studies are being conducted to investigate various fermenting microorganisms. Following enzymatic hydrolysis, the newly discovered oleaginous yeast *Rhodotorula mucilaginosa* KKUSY14 and *Lipomyces starkeyi* DSM 70,296 produced SCO from non-detoxified durian peel hydrolysates and paper mill waste, respectively [116, 117]. *Cryptococcus curvatus* produced significantly more lipids in the untreated wheat straw hydrolysate (33.5% w/w lipids per DW) than in the detoxified wheat straw hydrolysate (27.1% w/w lipids per DW), indicating that this oleaginous yeast is highly resistant to inhibitors. Although *Cryptococcus curvatus* demonstrated high resistance to furfural, 5-HMF, and acetic acid, it also demonstrated a high capacity for consumption of these inhibitory compounds in 24 h, as well as the ability to utilize furfural and acetic acid as carbon sources [118].

## Biomass degradation mechanisms in termite digestive tracts

**Fig. 12:**
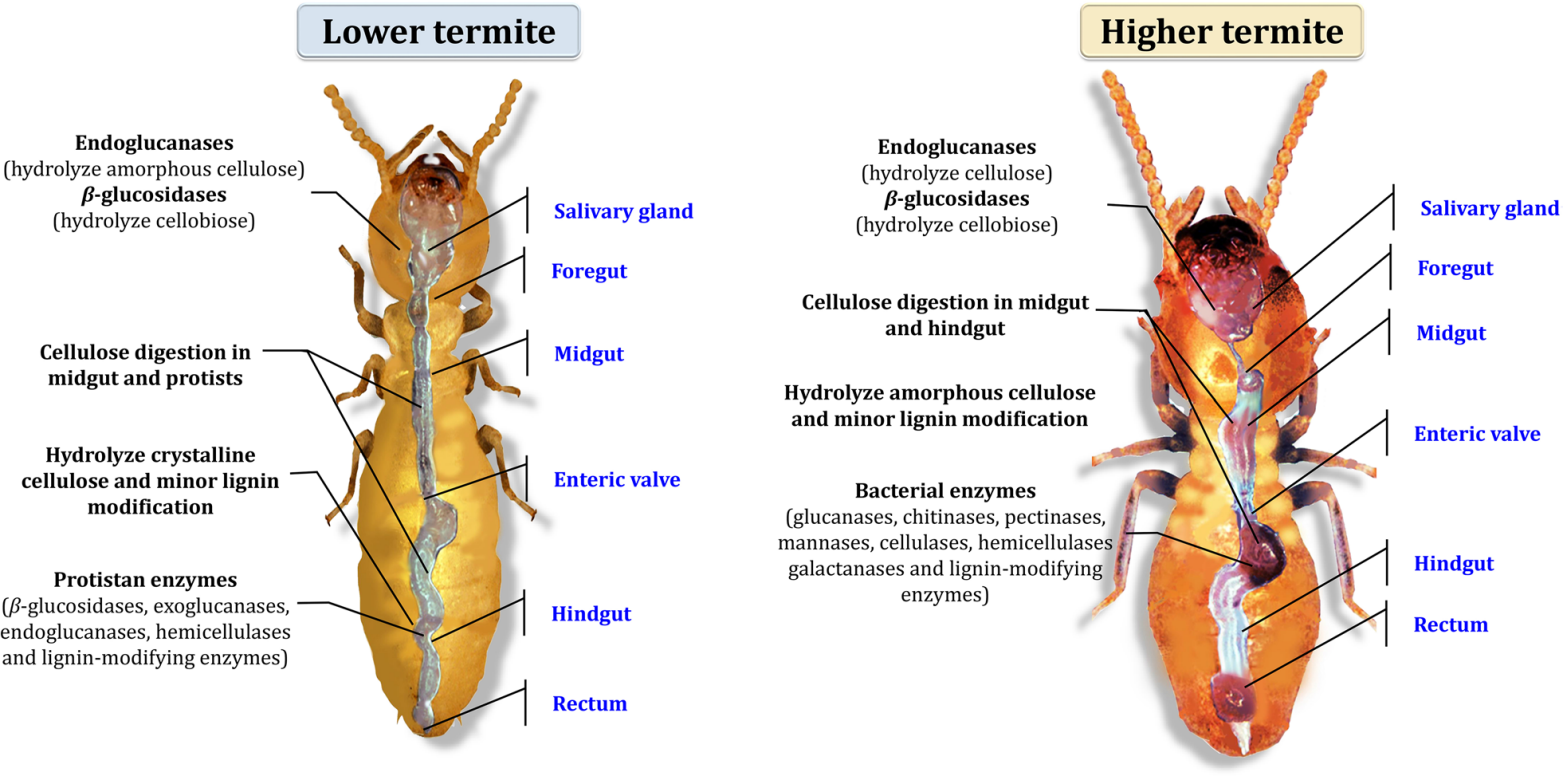
A diagram depicting the mechanisms of lignocellulose degradation in the digestive tracts of lower and higher termites. Adapted from Refs. [122, 123]

Termites consume a cellulose-rich diet, and digestion necessitates the presence of both the termite and its gut symbionts [119]. Lignocellulosic biomass is combined with the termite salivary gland enzymes, and partially digested biomass enter the termite digestive tract, where the gut symbionts hydrolyze the polysaccharides via enzyme secretion [120]. Termite gut symbionts secrete cellulases, hemicellulases, and lignin-modifying enzymes [121]. Figure 12 illustrates the lignocellulose degradation mechanisms in the digestive tracts of lower and higher termites [122, 123]. Hydrolysis of cellulose was initiated in the foregut of lower termites initiate cellulose hydrolysis in the foregut via salivary gland secretion of β-glucosidases and endoglucanases [124]. In the hindgut, symbiotic flagellated protists endocytose partially degraded food particles, hydrolyze crystalline cellulose and hemicellulose, and possibly modify lignin via the production of cellulases, hemicellulases, and lignin-modifying enzymes [125]. It is more complicated for higher termites to break down lignocellulose, and the process differs from one species to another. Endoglucanases are produced in the midgut of wood-feeding termites (*Nasutitermitinae*), whereas these enzymes are produced in the hindgut of *Termitinae* [126]. Endoglucanases, on the other hand, have been discovered in the midgut and hindgut of some *Nasutitermitinae* species, which is unusual [127]. The activity of β-glucosidases varies among different species of termites, as well. Some *Nasutitermes* species, for example, produce these enzymes from their salivary glands and the midgut, among other places [128]. The β-glucosidase and endoglucanase activities of *Macrotermitinae* can be found in both the salivary glands and the midgut of these termites. The β-glucosidase and endoglucanase activities of *Odontotermes formosanus* (Shiraki) are produced in the salivary glands [129], whereas *Macrotermes barneyi* secrete β-glucosidase and endoglucanase in the midgut [123]. As opposed to other subfamilies of higher termites, the activity of lignocellulose-degrading enzymes in *Macrotermitinae* is more complex, owing to the presence of basidiomycete fungi within the nest, which are distinguished by their lignocellulose degradation abilities [130]. When wood fragments are ingested by *Macrotermitinae*, it is believed that they combine with salivary β-glucosidase and endoglucanase and are then broken down into small particles in the foregut before moving to the midgut for digestion [131]. Endoglucanase and β-glucosidase enzymes are also secreted by the midgut, which act synergistically with salivary cellulases to hydrolyze amorphous regions of cellulose [132]. *Macrotermitinae* have also been found to have high levels of xylanase activity in their midguts [133]. Once partially digested food reaches the hindgut, it is attacked by lignocellulolytic enzymes secreted by the gut symbionts, resulting in food waste digestion and partial lignin modification [134].

## Azo dyes as aromatic textile wastewater pollutants

**Fig. 13:**
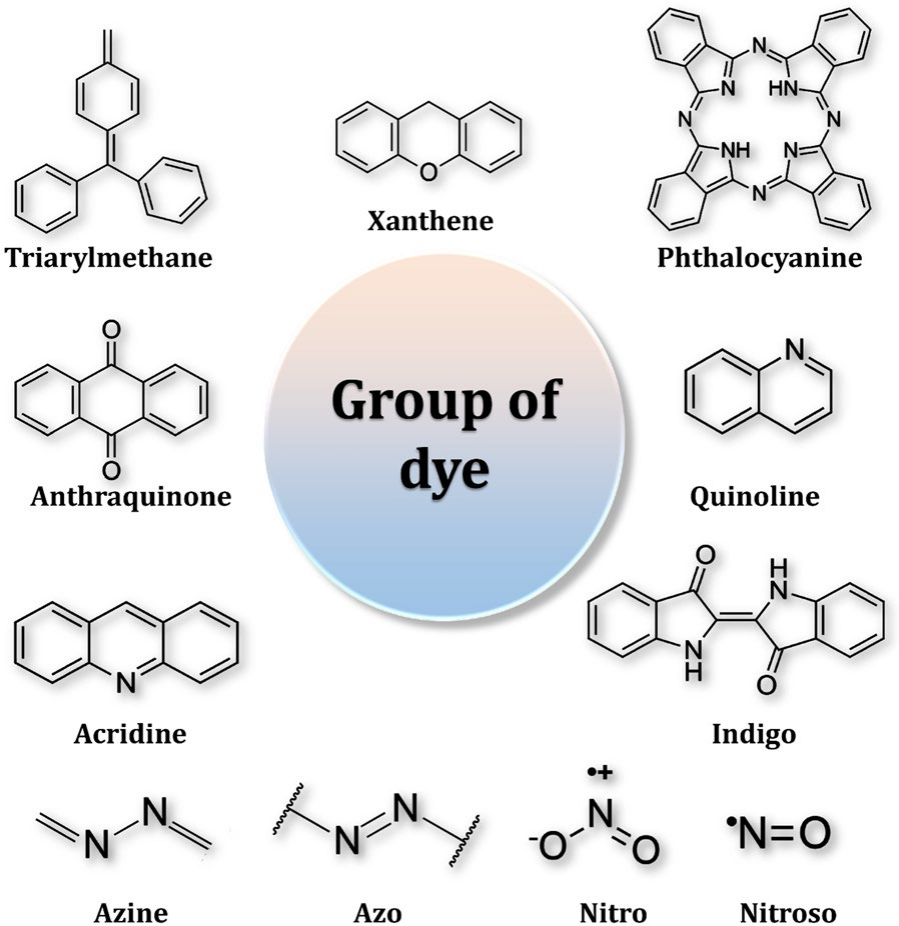
Chemical structure of textile dye groups

Dyes are chemical compounds that impart color to a substance [135]. Dyes are composed of two major components: (I) the auxochrome moiety, which determines their water solubility and binding to textile fibers, and (II) the chromophore group, which imparts color to fabrics [136]. The classification of textile dyes in Fig. 13 is based on their chemical structures and the color index system [137–143]. The majority of dyes contain chemical functional groups such as carboxylic, amine, or azo groups, which are occasionally conjugated to form aromatic structures [144]. The presence of more aromatic nuclei in the dye matrix enhances double bonds and molecule complexity [145]. Due to these incredibly complex structures, synthetic dyes typically exhibit extremely low degradability and recalcitrance [146]. The textile industry is a large consumer of water [147]. Additionally, the wastewater generated by this industry is a significant source of pollution due to its oxidative substances, persistent color, low biodegradability, and alkalinity [148]. Furthermore, the discharged liquid is a descendant runoff from a series of chemical baths that generates large volumes [149]. Textile dyes have a high molecular stability when subjected to chemical oxidation and light degradation. Furthermore, they may have biomagnification, toxicity, and even carcinogenic properties, all of which can be harmful to human health [15, 150, 151]. As a result, textile wastewaters are a source of concern and should be carefully treated to mitigate their environmental impact [152–155].

Numerous chemical and physical techniques, including Fenton oxidation, membrane filtration, ozonation, coagulation/flocculation, electrochemical treatment, advanced oxidation process, and adsorption, were investigated under this scope for the treatment of dye-containing textile wastewater [15, 156]. However, these methods are frequently not preferred due to a number of drawbacks, including toxic byproducts and sludge production, high costs, extensive infrastructure requirements, and limited applicability [15, 157]. Furthermore, the presence of high temperatures, chemical oxygen demand (COD), biological oxygen demand (BOD), pH, color, and heavy metals frequently impedes their use in textile wastewater treatment [158].

**Fig. 14:**
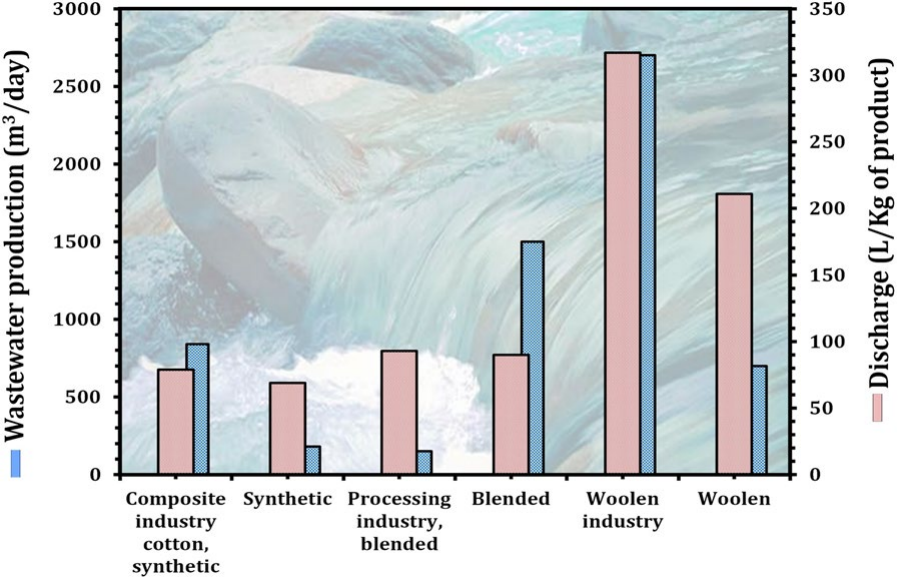
Quantity of wastewater produced by textile industries. Source of data from Refs. [163, 164]

The demand for fresh water is expanding quickly as a result of urbanization and industrialization, and is expected to exceed 55% by the year 2025 [159]. Recently, the "wastewater treatment field" has become a hot topic in both the academic and industrial communities, with the goal of removing chemical and microbiological pollutants from municipal/industrial wastewater [156]. Nonetheless, reusing treated effluent water can constitute a major health risk due to contamination from microbiological contaminants and organic matter. To date, biological treatment is widely recognized as one of the most environmentally friendly and cost-effective methods of removing toxins from wastewater [15, 160]. Notably, the use of oleaginous microorganisms in biological wastewater treatment is far more appealing than traditional aerobic and anaerobic digestion technologies, which necessitate sophisticated systems [161]. While oleaginous microorganisms can be cost-effective, they also have the ability to quickly clean wastewater and produce high-value-added products [162]. Utilizing oleaginous microorganisms in low-cost substrates such as nutrient-rich wastewaters is clearly a circular economy concept that has the potential to sustain industries associated with wastewater treatment plants. Textile industry consumes a significant amount of potable water in the production of fibers, resulting in massive amounts of wastewater (Fig. 14) [163, 164]. One kilogram of textile material requires approximately 200 L of water to manufacture. This quantity of water is required when chemicals are applied to fabrics and when the finished products are rinsed. China exports the most textiles of all kinds, followed by the European Union, India, and the United States [165].

## Azo dye classification and toxicity

**Fig. 15:**
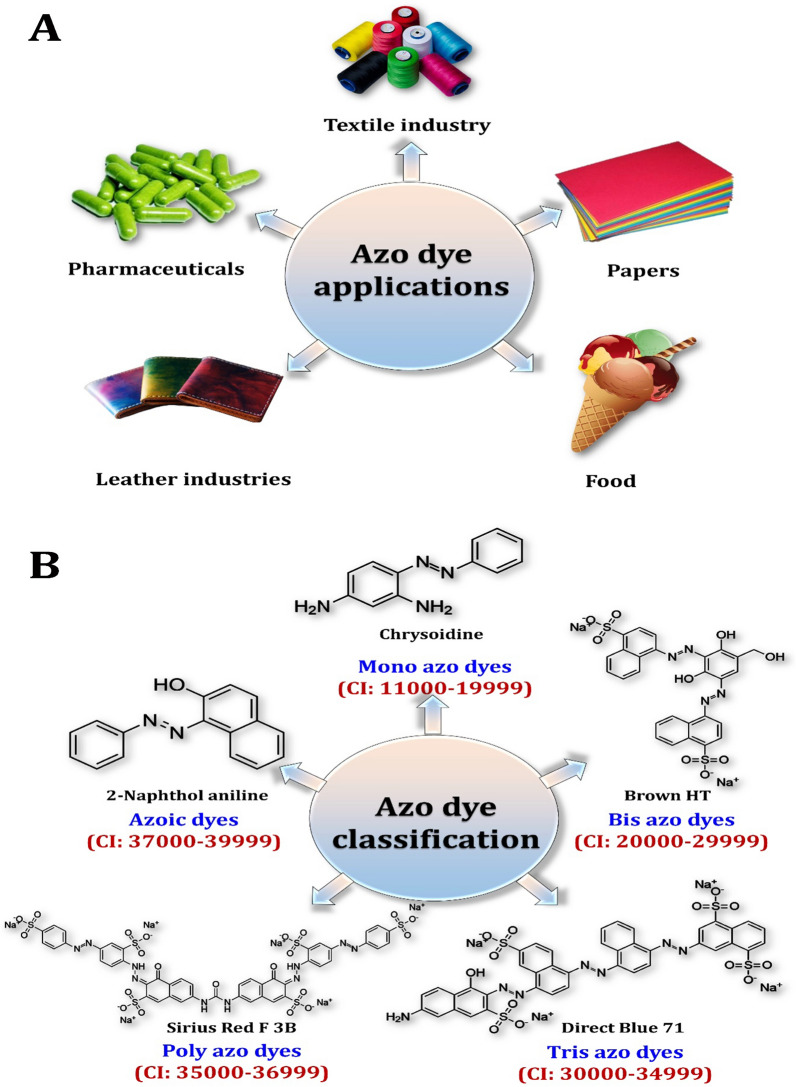
Applications of azo dyes (**A**) and their classification (**B**)

Azo dyes are among the most hazardous types of synthetic dyes produced in textile manufacturing effluents due to their chemical structure, which contains azoic linkages, amino groups, and aromatic rings. The global annual production of synthetic textile dyestuff is estimated to be over 1 × 10^6^ ton [166]. Azo dyes account for more than 70% (9,000,000 tonnes) of all synthetic dyestuffs and global manufacturing needs [167]. In comparison to natural dyes, azo dyes are more widely used in textile, pharmaceutical, and other industries (Fig. 15A) due to their ease of manufacture, resistance to fading, and color diversity. Figure 15B depicts the classification of azo dyes. According to the radical group attached to the azo bond, azo dyes are classified into two groups: the first group contains a radical attached to an aromatic amine moiety (NH_2_–R–N=N–R′), whereas the second group contains only radical compounds (R–N=N–R′) and generates aromatic amines upon reduction [145]. While reduction is the predominant reaction type involved in the degradation of the majority of azo dyes by specific enzymes called azo reductases, a few dyes are cleaved via oxidation using oxidases and peroxidases [145].

Azo dyes and their metabolites containing AAs are toxic, carcinogenic and mutagenic [168]. It has been reported that 15–50% of azo dyes are deposited in wastewater, which is frequently used for irrigation in developing countries [169]. Azo dyes also alter soil chemistry, disrupting the microbial soil balance and impairing plant germination and flowering [145]. Azo dyes found in textile industry effluents can cause environmental stress and increase abscisic acid secretion in plants, impairing their normal growth [170]. Surprisingly, azo dyes from textile effluents were found in contaminated plants, where they wreaked havoc on plant physiology and growth [171]. It has been demonstrated that the azo dye Black Dye Commercial Product (BDCP) is both an aneugen and a clastogen, causing chromosomal aberrations, micronuclei formation, and cell death in *Allium cepa* meristematic cells after exposure to 1.0 ppb of BDCP [172]. The biodegradation of BDCP by a consortium of diverse bacterial strains and yeast revealed that the toxic metabolites were more cytotoxic and genotoxic than the parent compound. The AAs produced by the bacterial and yeast azo reductases upon azo bond cleavage are most likely the cause of the azo metabolites' more adverse effects [25, 26, 173]. Water coloring is caused by the release of textile effluents, particularly red, orange, and yellow azo dyes, into the water, thereby reducing sunlight penetration and thus plant photosynthesis and growth rate [15]. Additionally, azo dyes dispersed in water provide an abundant substrate for bacterial populations and aquatic microorganisms. The AA precursors formed during the biodegradation of azo dyes contribute to the overall increase in the water content of toxic amines, which have a more detrimental effect on fish and aquatic organisms.

**Fig. 16:**
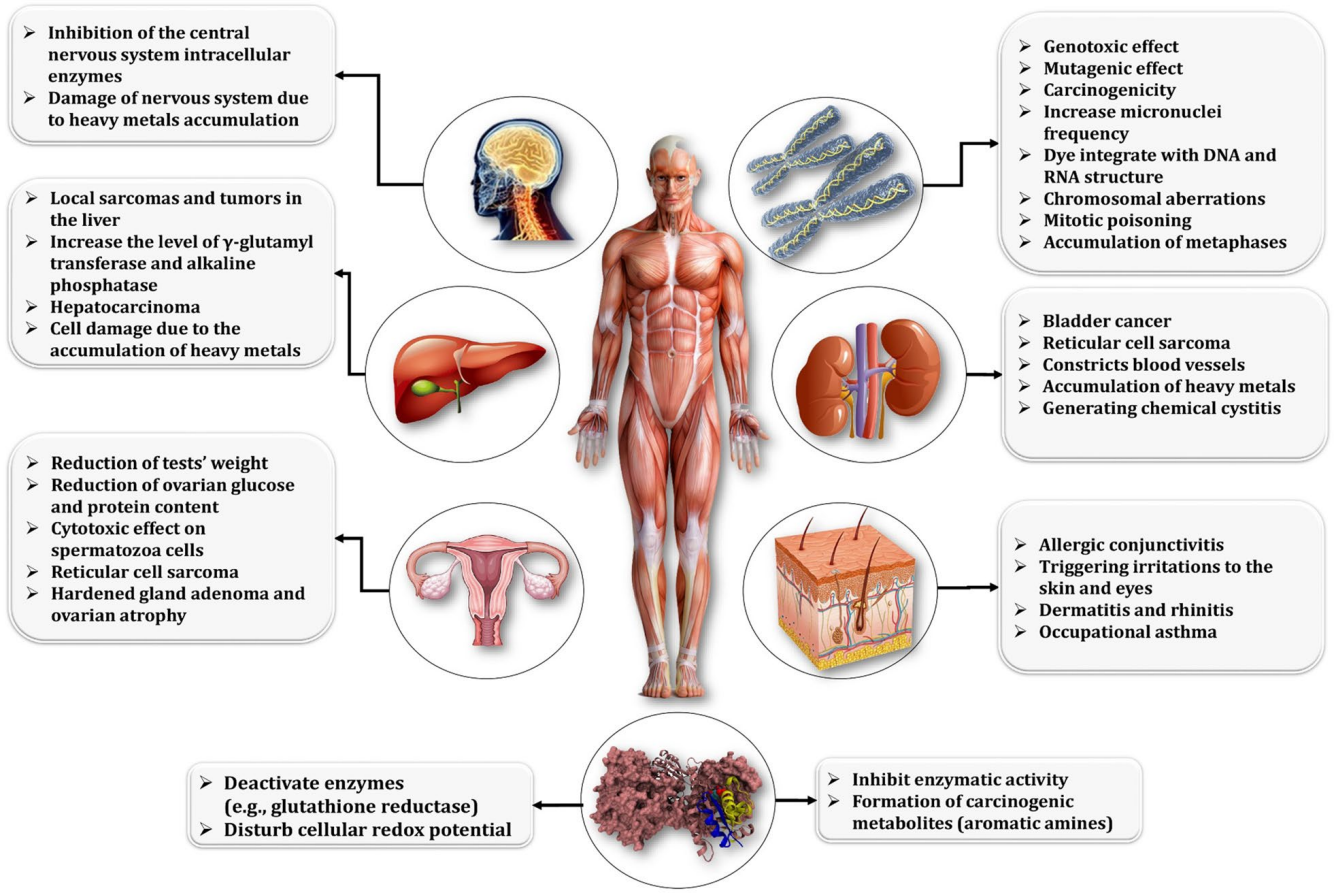
Impact of azo dye on humans

Azo dyes and their degraded byproducts also have detrimental effect on humans (Fig. 16). The AAs formed as a byproduct of azo dyes have the potential to be carcinogenic. Numerous symptoms have been observed in zebrafish when exposed to azo dye at concentrations ranging from 5 to 50 mM, including spinal deformities, placenta swelling, and heart swelling [174]. The most common routes of exposure for humans to azo dyes are inhalation, skin contact, and oral ingestion. Allergic conjunctives and rhinitis are the initial symptoms that affect workers and typically occur prior to the onset of occupational asthma [175]. Allergic conditions develop as a result of histamine's association with immunoglobulin (IgE) antibodies produced against circulating antigens that were previously formed by the reaction of reactive azo dyes with human serum albumin [176]. The skin is a critical route for xenobiotics to enter the human body. *Corynebacterium*, *Enhydrobacter*, *Micrococcus*, *Propionibacterium*, *Staphylococcus*, *Streptococcus*, and *Veillonella* are the most abundant bacterial strains physiologically present on human skin [177]. The majority of these dermal species cleave azo dyes, such as Orange II and Methyl Red, into toxic AAs, which may be absorbed directly into the skin tissue and induce carcinogenesis [178]. Additionally, dermal mutagenesis was induced by direct contact with 40 mutagenic AAs derived from 180 parent azo dyes used in the textile industry [178].

Consumption of azo dye-contaminated water can result in acute toxicity symptoms such as irritation, inflammation, or system failure. Crystal Violet is an azo dye that may be present in textile industry wastewater, causing irritation of the gastrointestinal tract, inflammation of the urinary bladder, and kidney failure [179]. Chronic exposure to azo dyes or their AAs byproducts can initiate carcinogenesis. Disperse Red 1, an azo dye widely used in textile coloring, increased the occurrence of micronuclei in human hepatoma cells and lymphocyte culture in vitro, while the damage caused by Disperse Red 1 established its clastogenic effect on chromosomes [180]. Additionally, when the Disperse Red 1 dye was tested in vivo on mature male mice, it induced testicular cytotoxicity, abnormal sperm morphology, and genotoxic DNA damage to spermatogonia in the testes [181]. Azure-B, another widely used azo dye in the textile industry, influenced human behavior by inhibiting monoamine oxidases A in the nervous system [182]. Human and other mammalian intestinal microflora anaerobically degrade azo dyes to their AAs, resulting in carcinogenic nodules in the liver, as well as breast and hematopoietic cancer [183]. The AAs degraded by the intestinal microbiome are diverse in general. For example, the Sudan II azo dye, which is widely used in the textile industry, can be biodegraded to a variety of metabolites, including 2,4 dimethylaniline AA. After three hours of post-treatment with 2,4 dimethylaniline via gastric gavage, damaged DNA was detected in the lungs, kidney, and liver tissues [184]. It has been reported that *p*-phenylenediamine can cause acute kidney failure in humans. Additionally, O-Toluidine can cause DNA damage and aneuploidy in cultured mammalian cells [185]. Benzidine, the reduction product of Congo Red and other azo dyes, is well-known for its carcinogenicity to various organs and cells in the body, including the stomach, large intestine, liver, gall bladder, bile duct, and pancreas. Additionally, it has the potential to cause cancer in the respiratory and genitourinary systems, most notably in the lungs and kidney cells [186]. Notably, when benzidine is released into water following the treatment of textile effluents, acute oral toxicity symptoms such as nausea, dizziness, headache, cyanosis, and mental confusion can manifest [187].

## Mechanisms by which yeasts degrade azo dyes

While yeasts have several advantages over filamentous fungi and bacteria when it comes to degrading textile dyes, their ability to develop rapidly and tolerate stressful conditions are two of the most significant advantages [24]. Only a few reports, however, have highlighted the degradation and removal of azo dyes by yeasts. Several yeast strains, including *Pichia occidentalis*, *Scheffersomyces spartinae*, and *Sterigmatomyces halophilus*, have been shown to be capable of degrading a variety of textile dyes, including azo dyes, as well as to withstand severe conditions, such as high salt concentrations in textile wastewater [25, 26, 188]. The ability of *Saccharomyces uvarum*, *Torulopsis candida*, and *Saccharomycopsis lipolytica* to biosorb Reactive Brilliant Red K-2B was determined [189]. *Saccharomyces cerevisiae* was also evaluated for its ability to remove Remazol Blue, with results indicating that biosorption treatment can reduce the COD value and color absorbance of wastewater containing Remazol Blue dye by 61.8 and 100%, respectively [190].

Regarding the biodegradation mechanism, reductive or oxidative reactions are commonly involved in the biodegradation of azo dyes using a yeast-mediated enzymatic system. Lignin-modifying enzymes have a high potential for decolorizing and degrading azo dyes used in textiles [28]. In general, reductive reactions result in the azo dyes cleaving of AAs into less toxic compounds that mineralize into CO_2_ and H_2_O. Azoreductase and NADH-dependent reductases are putatively involved in this phase [26]. Yang et al. [191] demonstrated the efficiency of Reactive Black 5 degradation by manganese peroxidase-producing *Debaryomyces polymorphus*, whereas Jadhav et al. [192] found an increase in lignin peroxidase and other enzyme activity in *Saccharomyces cerevisiae* during Methyl Red decolorization. Qu et al. [193] described the mechanism by which yeasts remove azo dyes, which included asymmetric azo bond cleavage, adsorption, and hydroxylation. The dye is first converted to AAs such as 3-amino-4-hydroxy-naphthalene-1-sulfonate and 4-amino-naphthalene-1-sulfonate, which are then oxidized to ortho-hydroxyl compounds. In general, ligninolytic enzymes oxidize azo dyes to form a carbonium ion, which then undergoes nucleophilic water attack to form a diazene derivative and a benzoquinone. Following diazene oxidation, molecular nitrogen is liberated, resulting in the formation of hydroperoxide derivatives.

## Biodiesel (lipid) production

Biodiesel production by several oleaginous microorganisms, such as microalgae, fungi, and bacteria, have been extensively studied [19, 194–196]. Lipids mainly accumulate in the TAG form, polar lipids, and free fatty acids in cell biomass. Oleaginous microorganisms have been defined as organisms with a lipid content exceeding 20% of total biomass [2]; therefore, they can be considered as sustainable biofuel precursors. Especially yeasts represent promising lipid producers among the oleaginous microbes, as they are characterized by a high growth rate, they have a high lipid yield and are also able to use low-cost substrates such as agricultural residues, resulting in high content of C16–C18 fatty acids [2, 18, 75, 197].

## Biosynthetic pathways of lipids by oleaginous yeasts for biodiesel production

**Fig. 17:**
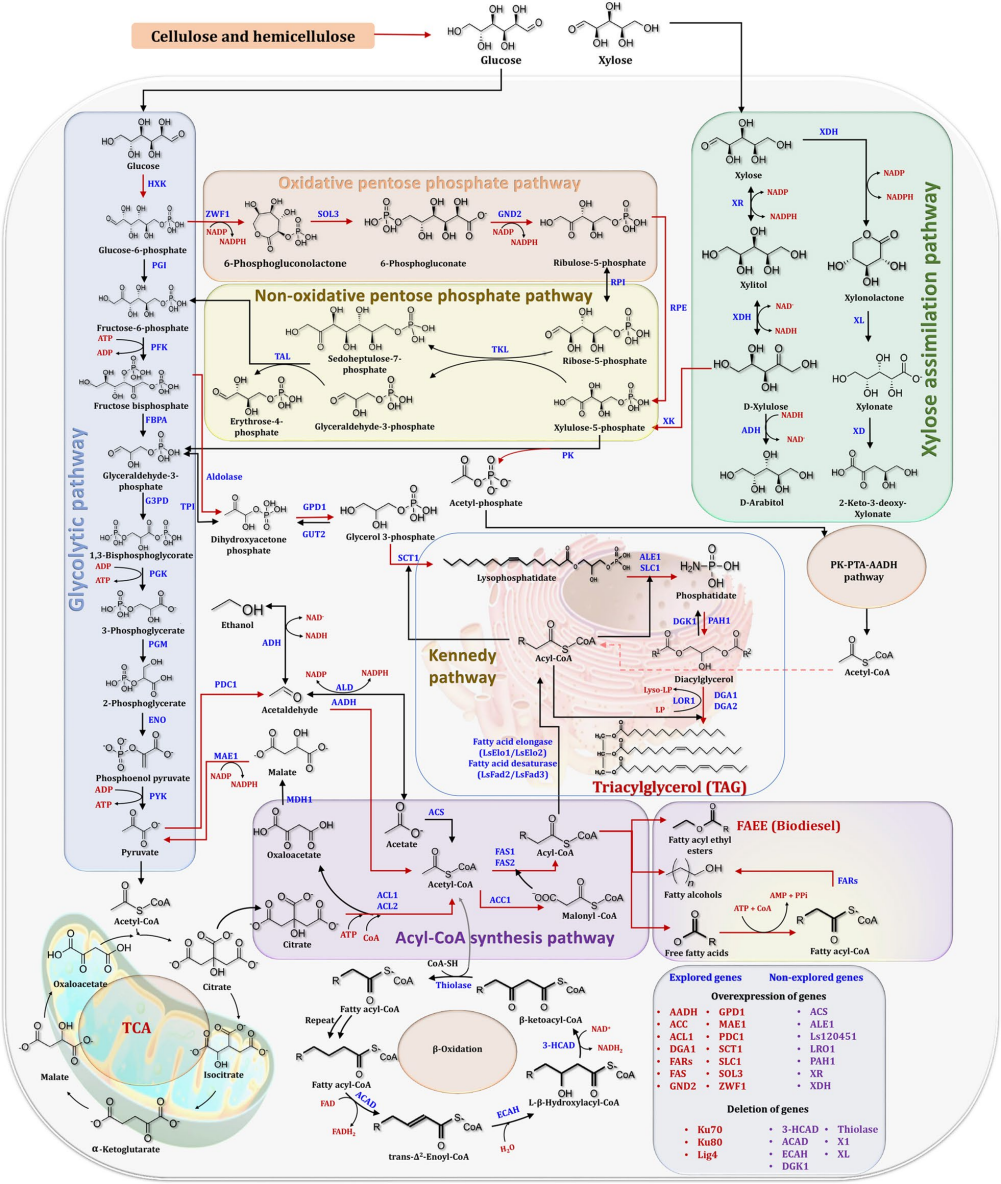
TAG synthesis pathways in yeast using glucose and xylose as carbon sources, as well as the explored (red) and unexplored (violet) genes encoding enzymes. Adapted from Refs. [72, 83, 198, 199]

Figure 17 depicts the biosynthetic pathways for lipids by oleaginous yeasts, which are based on previously reported key enzymes and genes [72, 83, 198, 199]. In oleaginous yeasts, lipids are synthesized via two distinct biosynthetic pathways. Lignocellulosic biomass sugars are used as a hydrophilic carbon substrate in the de novo pathway [200]. However, oils are used as a hydrophobic carbon substrate for lipid biosynthesis in the ex novo pathway. Acetyl-CoA and nicotinamide adenine dinucleotide phosphate (NADPH) are required for the de novo lipid biosynthesis pathway. Acetyl-CoA is synthesized from fatty acids, sugars, and amino acids, whereas NADPH is synthesized via the pentose phosphate pathway [201]. A condition in which the fermentation broth contains an excess of carbon and a deficiency of nitrogen can result in lipid synthesis [202]. As illustrated in Fig. 17, the majority of the enzyme-catalyzed reactions involved in the Kennedy pathway's lipid production have been identified. *Lipomyces starkeyi* exhibited some novel lipid accumulation pathways in comparison to other lipid-producing yeasts such as *Rhodotorula glutinis*, *Yarrowia lipolytica*, and *Rhodosporidium toruloides* [203]. Due to the fact that *Yarrowia lipolytica* was considered a degrading yeast, metabolic engineering operations were required to achieve efficient lipid accumulation in this wild-type yeast [204]. In comparison to non-oleaginous yeasts, the oleaginous yeast *Lipomyces starkeyi* was unique in the citrate/malate shuttle for citrate transport and the pyruvate dehydrogenase pathways [198]. Additionally, yeast fermentation can be used to utilize xylose, which is the second most abundant sugar in biomass hydrolysate. It was previously reported that xylose utilization was significantly lower in wild-type *Lipomyces starkeyi* than glucose utilization [205]. This indicated that an increase in the rate of xylose metabolism was required via metabolic regulation mechanisms. The carbohydrate xylose can be converted to D-xylulose, which can then be converted to xylulose 5-phosphate, then be converted to acetyl phosphate via the enzyme phosphoketolase, which finally enters the PK-PTA-AADH pathway and contributes to lipid synthesis (Fig. 17).

## Biodegradation pathways of lignin-based aromatics by yeasts inhabiting termite guts and biodiesel production

**Fig. 18:**
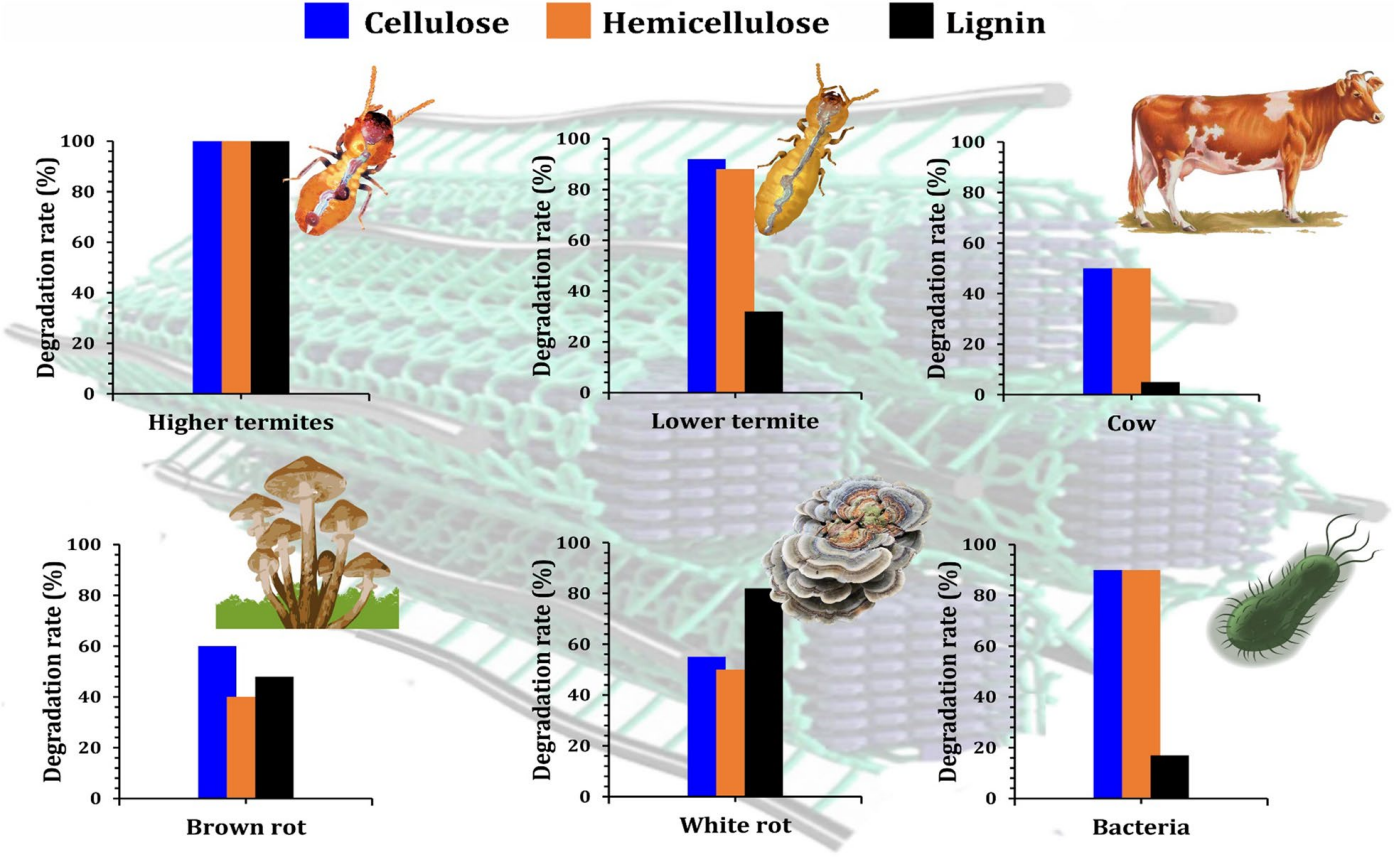
Representative lignocellulolytic systems and their efficiency of conversion for the major biomass polymers (cellulose, hemicellulose, and lignin)

**Fig. 19:**
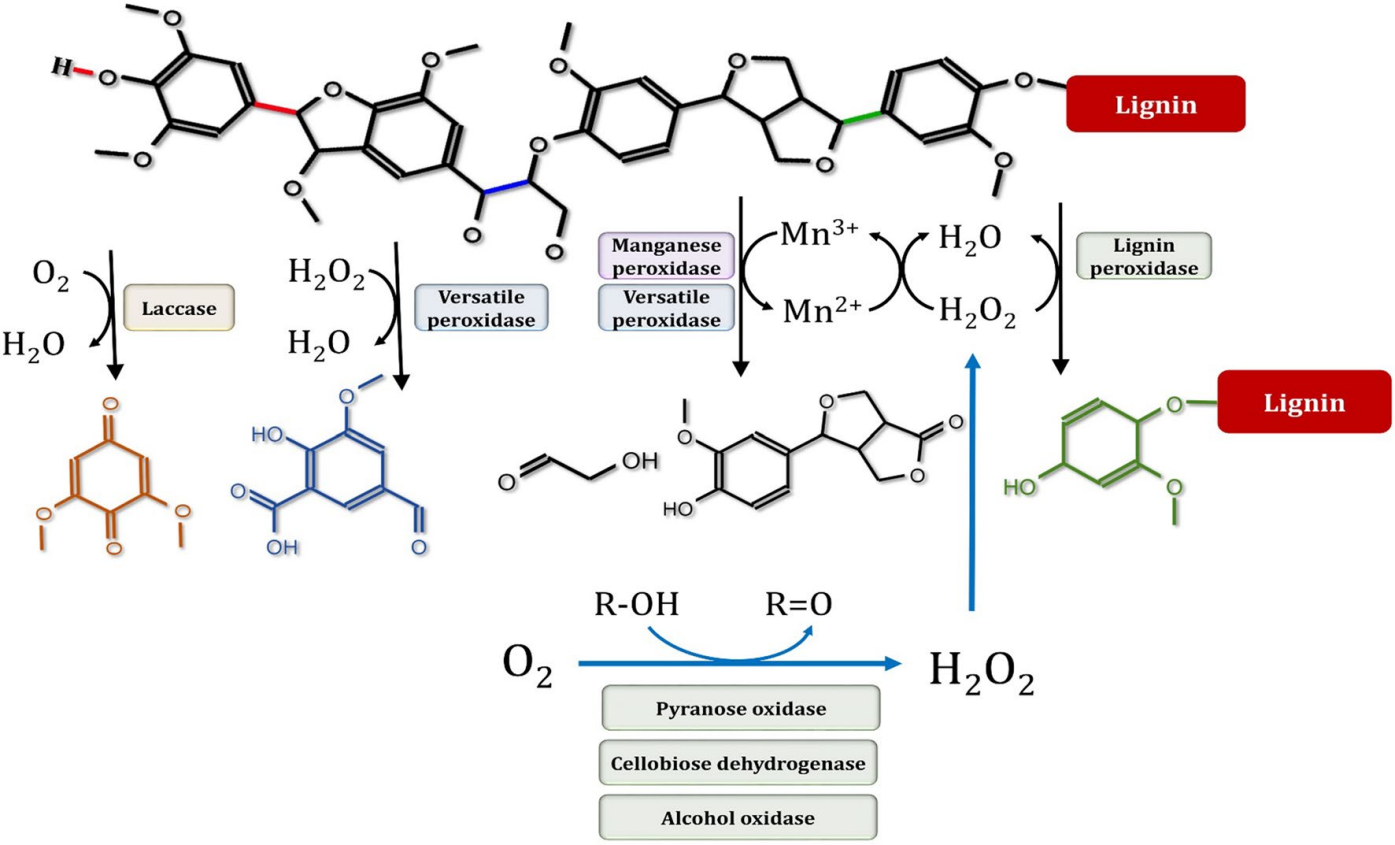
A representative diagram of bond cleavage catalyzed by various ligninase families

Among various organisms, termites represent the most efficient lignocellulose-degrading systems for the three main polymers of biomass namely, cellulose, hemicellulose and lignin (Fig. 18). The degradation of lignin by wood-feeding termites is fascinating, and while much evidence has recently been gathered, several critical processing characteristics remain ambiguous. A termite gut system is generally considered an anaerobic environment due to the large community of strictly anaerobic microorganisms such as yeasts found in the termite guts. For lignin degradation, laccase and phenol-oxidase gene expression, as well as pyrogallol oxidation activity, were confirmed to occur in the salivary glands of the termite, *Reticulitermes flavipes* [22]. In order to decompose lignin and assimilate its aromatic building blocks, yeasts have evolved a number of sophisticated metabolic pathways. Several laccases and peroxidases are used to break different linkages connecting the S, G, and H-lignin monomers in these metabolic pathways, which are required for lignin degradation (Fig. 19) [206]. Glutathione S-transferases, etherases, thiolases, and cytochrome P450 have also been linked to lignin depolymerization. *Cutaneotrichosporon oleaginous* ATCC 20,509 can grow on lignin, can tolerate high concentrations of 15 different monoaromatic compounds, and can accumulate lipids in excess of 69% of dry weight [207]. The strain's tolerance for stirring and several inhibitors, as well as their metabolic flexibility, reflect their ability to utilize undefined and impure lignin feedstocks in nonsterile conditions, thereby lowering the process's cost. *Rhodosporidium toruloides* DSM-4444 and IFO 0880 are two additional yeasts that have been shown to metabolize aromatic lignin monomers [207, 208]. Only *Rhodosporidium toruloides* DSM-4444, on the other hand, was able to metabolize the four models of lignin monoaromatics using lignin as the sole carbon source [208]. The advancement of multi-omics technology has increased accessibility to lignin-related metabolic pathways. Clearly, yeasts and fungi depolymerize lignin and lignin-based aromatic compounds more efficiently than bacteria [209]. When lignin depolymerizes to monomers and/or low-molecular-weight aromatics, yeasts use them to assimilate carbon and energy through their well-adapted metabolic pathways.

## Pathways for lignin-based aromatics degradation by yeast inhabiting termites and biodiesel production

Termites, an influential group of social insects known for destroying human property, are now considered an informative bioreactor that can efficiently degrade lignocellulosic biomass [210]. Indeed, termites contribute significantly to the global carbon recycling process occurring within natural ecosystems [211]. Their capacity to dissipate 74–99% of the cellulose and 65–87% of the hemicellulose they consume is remarkable, allowing them to use the material as their primary source of energy [24]. Termite digestome "termites and their gut symbionts" is an ideal biological model for improving the current inefficient biorefinery processing of lignin-based aromatic wastes due to their ability to thrive on recalcitrant lignocellulosic biomass and their widespread distribution [24].

**Fig. 20:**
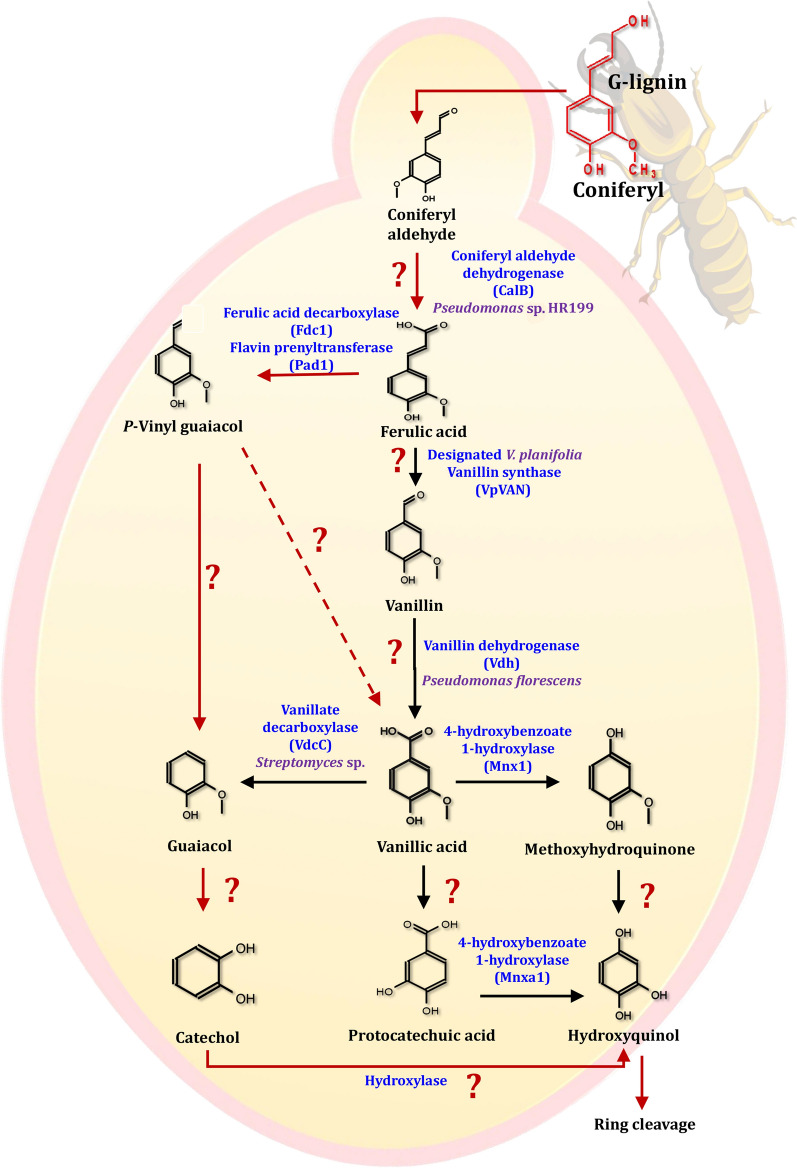
The scheme of G-lignin-based aromatics degradation pathways in yeasts. The red lines represent the potential ferulic acid pathway. The use of question marks indicates that the enzyme has not been identified. The suggested gene, which encodes for specific enzymes, was found in the mentioned species

Lignin polymer networks are primarily composed of *p*-hydroxyphenyl (H), guaiacyl (G), and syringyl (S) units, which are formed by dehydrogenating and polymerizing three different hydroxycinnamyl alcohols (monolignols), namely *p*-coumaryl alcohol, coniferyl alcohol, and sinapyl alcohol [22]. The G-lignin unit comprises 37.8 and 98.3% of the lignin in poplar (a typical hardwood) and pine (a typical softwood), respectively [212]. Ferulic acid has been used as a model compound for G-lignin for many years. It is structurally connected to the C-5 of the l-arabinofuranosyl residue, which is attached to the xylan backbone and acts as a lignification anchor in herbaceous biomass [213]. Although it is known that coniferyl aldehyde is converted directly to ferulic acid in the yeast *Saccharomyces cerevisiae*, the enzymes responsible for this conversion are unknown [214]. As a result, a set of candidate genes and degradation pathways for G-lignin-derived aromatics have been proposed in yeasts (Fig. 20). According to the literature, the yeasts *Brettanomyces anomalus*, *Saccharomyces cerevisiae*, and *Debaromyces hansenii*, all convert *p*-vinyl guaiacol to vanillin [215–217]. The conversion of ferulic acid to vanillic acid has been observed in the oleaginous yeasts *Cryptococcus curvatus*, *Trichosporon guehoae*, and *Rhodosporidium toruloides*, but the exact mechanism is unknown [208]. It has been reported that *Candida parapsilosis* demethylated vanillic acid via a non-heme iron monooxygenase mechanism [218]. In conclusion, vanillic acid and vanillin are significant G-unit monolignols because they were detected during the degradation of lignin [219]. Vanillin can be converted by yeast in two pathways, depending on the strain: as vanillic acid or as vanillyl alcohol. Before being cleaved from the ring, it is converted into three major compounds: protocatechuic acid, catechol, and hydroxyquinol. While yeasts and fungi frequently possess a pathway leading to hydroxyquinol, bacteria do not [220]. As a result, demethylation of vanillic acid via a non-heme iron monooxygenase mechanism is likely to be a major pathway in yeast and bacteria, whereas demethylation of vanillic acid to protocatechuic acid (PCA) is the primary pathway in filamentous fungi.

**Fig. 21:**
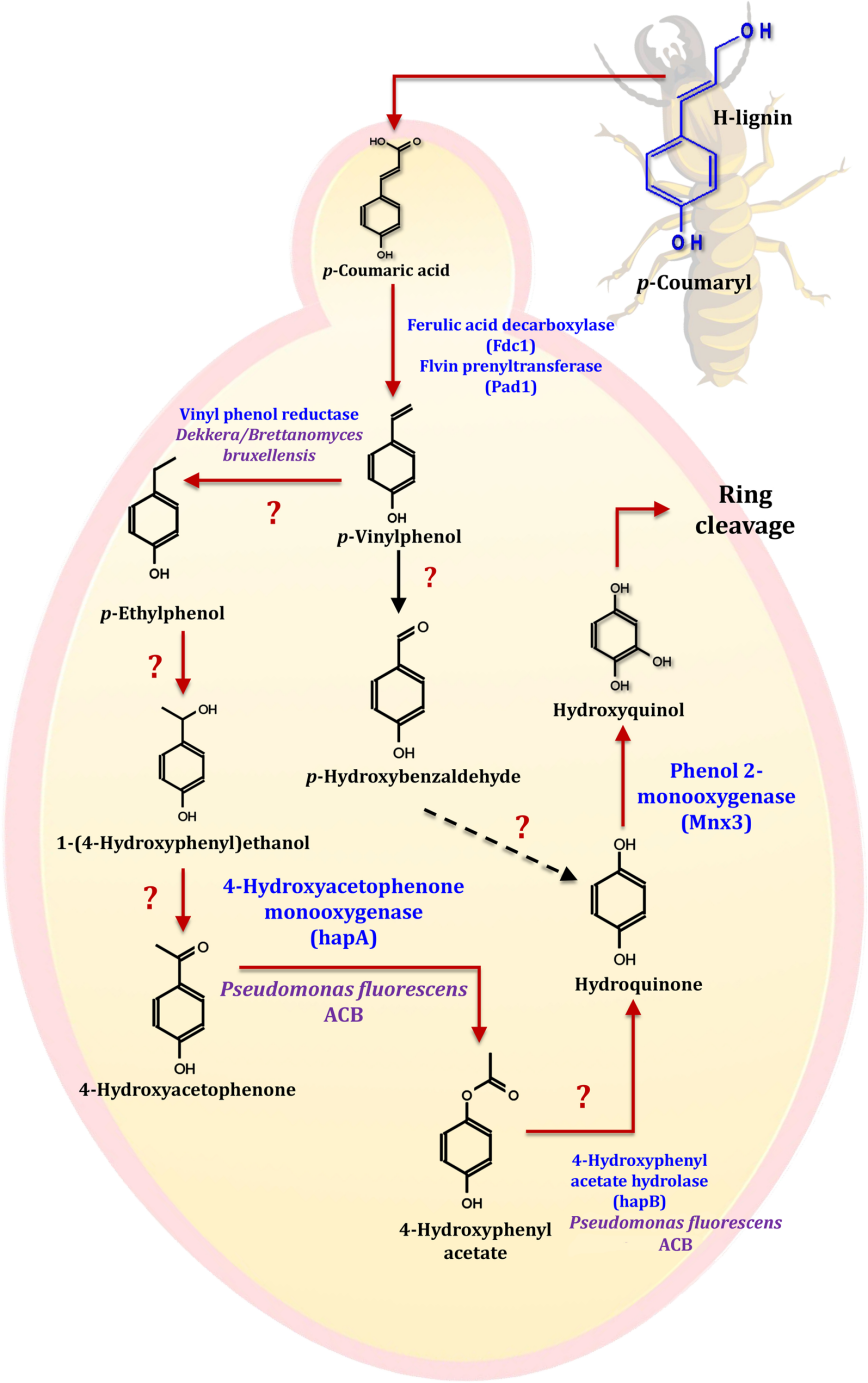
The scheme of H-lignin-based aromatics degradation pathways in yeasts. The prospect pathway for *p*-coumaric acid is represented by red lines. The use of question marks indicates that the enzyme has not been identified. The suggested gene, which encodes for specific enzymes, was found in the mentioned species

**Fig. 22:**
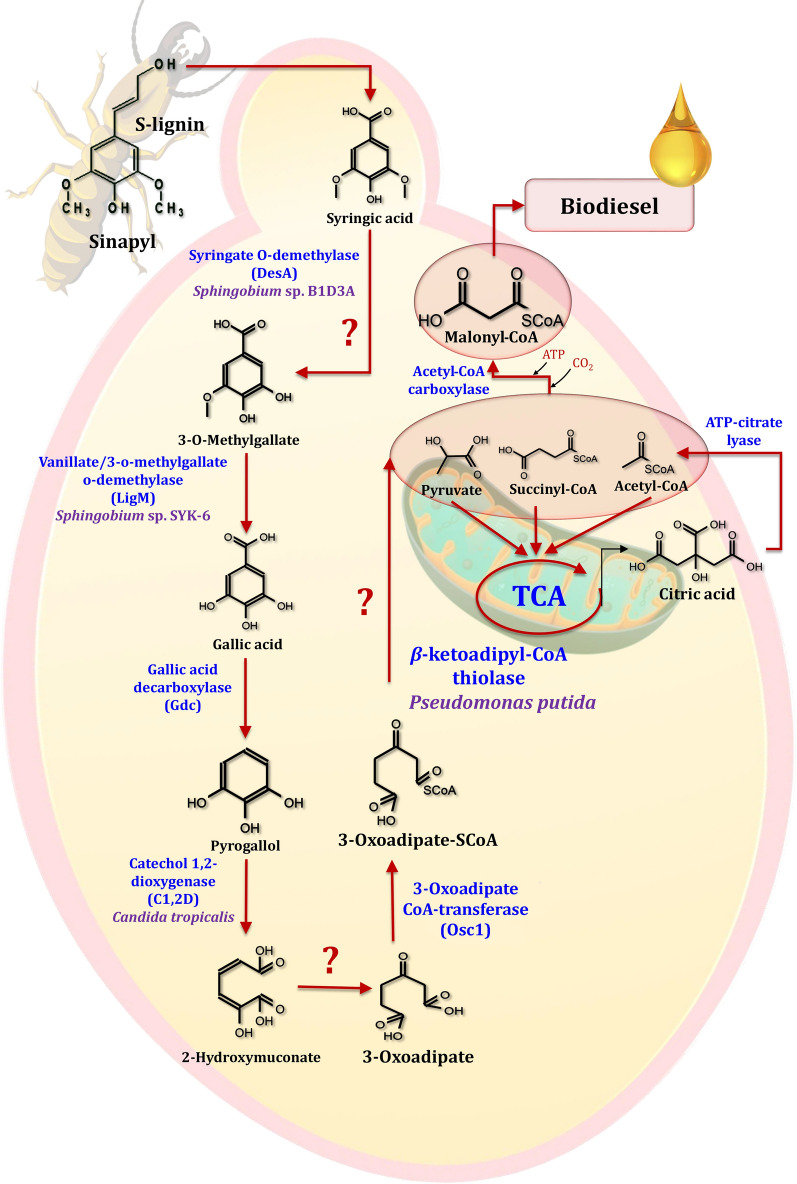
The scheme of S-lignin-based aromatics degradation pathways in yeasts. The prospect pathway for *p*-coumaric acid is represented by red lines. The use of question marks indicates that the enzyme has not been identified. The suggested gene, which encodes for specific enzymes, was found in the mentioned species

When it comes to poplar and pine wood, the H-lignin unit accounts for 0.3 and 1.7% of the total lignin content, respectively [212]. Pei et al. [221] reported that *p*-coumaric acid, a derivative of the monolignol *p*-coumaryl alcohol and a ferulic acid homologue, can be linked to polysaccharides and has antioxidant and antimicrobial properties. Cinnamic acid is converted to *p*-coumaric acid during the synthesis of lignin in plants [222]. Figure 21 depicts the degradation pathways for H-lignin-based aromatics in yeast. Suárez et al. [223] found that the yeast genera *Brettanomyces* and *Dekkera* were capable of converting *p*-vinylphenol into *p*-ethylphenol. However, no evidence has been found for the conversion of *p*-coumaric acid to *p*-hydroxybenzoic acid in the presence of yeast [214]. On the other hand, in poplar and pine wood, the S-lignin unit accounts for 61.9 and 0.0% of the lignin, respectively [212]. In comparison to G- and H-lignin, which contain one and zero methoxy groups on the aromatic ring, S-lignin contains two methoxy groups. As a result, degrading S-lignin is more difficult than degrading G- or H-lignin. Due to the structure of syringic acid, it is regarded as a model compound for S-lignin. Sinapic acid, a derivative of the monolignol sinapyl alcohol, is an aromatic compound with poorly understood aromatic metabolic pathways. In yeast, aromatic compounds derived from S-lignin are degraded via the pathways depicted in Fig. 22.

**Fig. 23:**
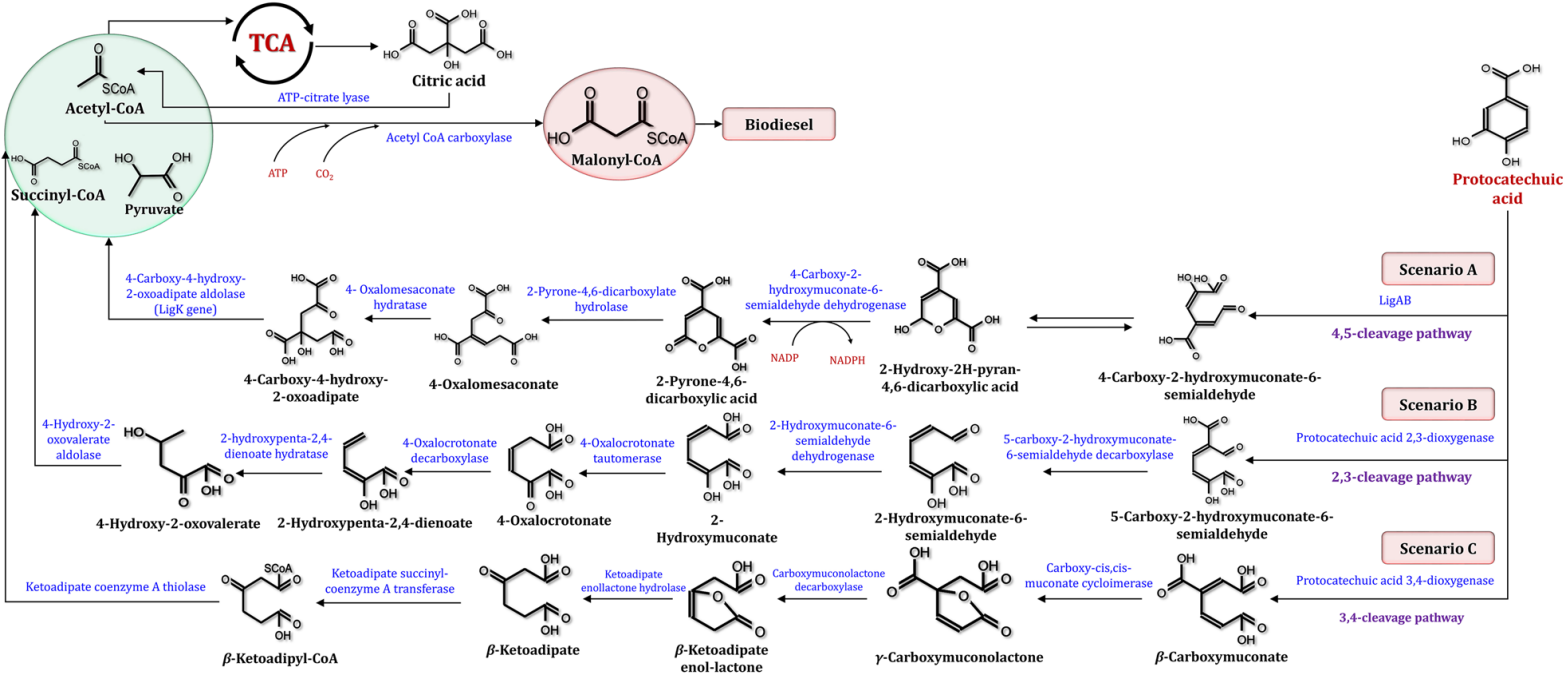
The proposed PCA biodegradation pathway during the production of biodiesel and other byproducts

**Fig. 24:**
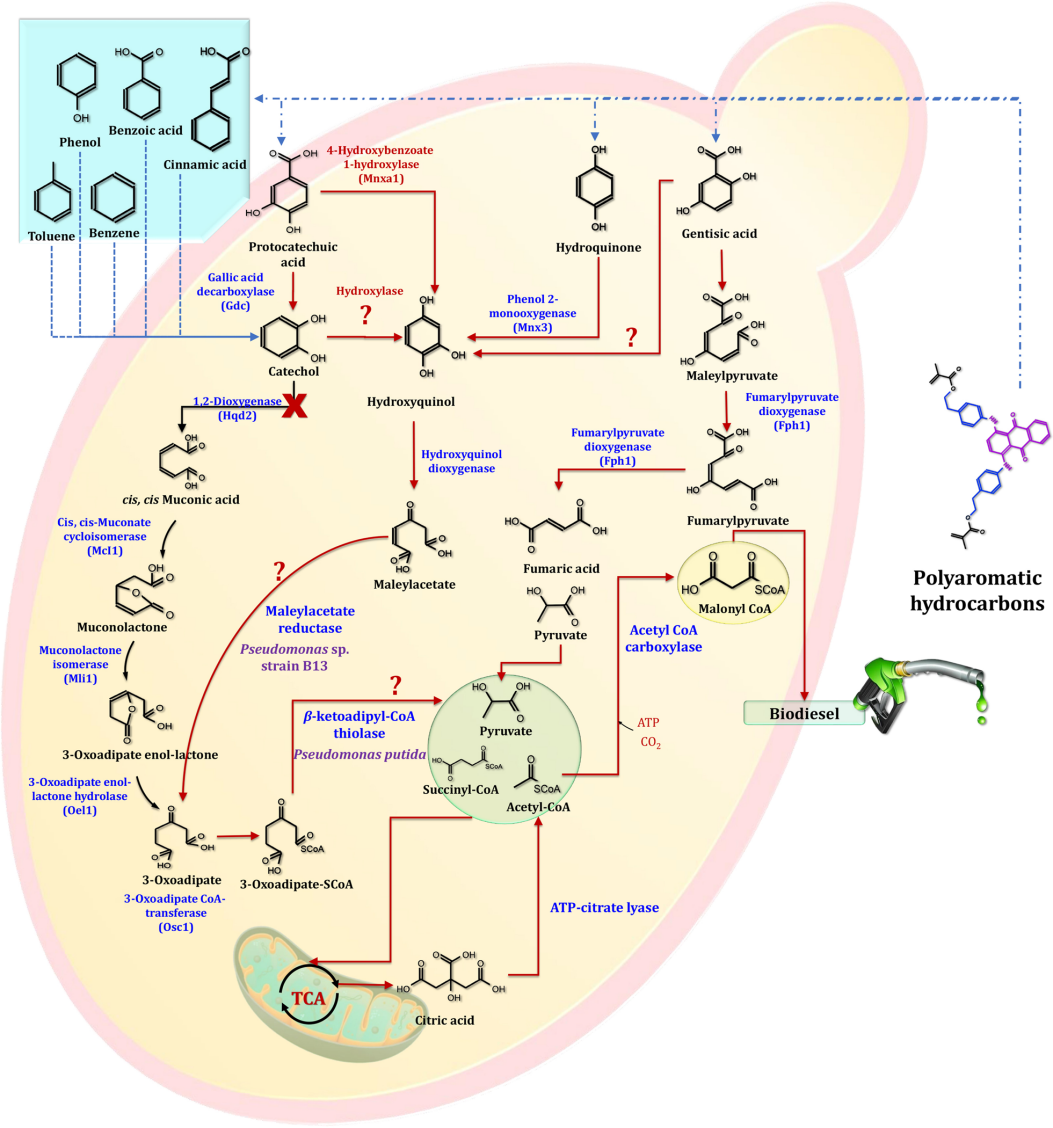
The scheme of PCA and catechl degradation pathways in yeasts. The prospect pathway for *p*-coumaric acid is represented by red lines. The use of question marks indicates that the enzyme has not been identified. The suggested gene, which encodes for specific enzymes, was found in the mentioned species

PCA and catechol are abundant in a variety of lignin hydrolysates. As can be seen, G- and H-lignin components are metabolized via the PCA 4,5-cleavage pathway, while S-lignin components are degraded via the PCA 4,5-cleavage pathway. As illustrated in Fig. 23, the degradation of PCA in microbes has been classified into three pathways: 3,4-cleavage [224], 4,5-cleavage [225], and 2,3-cleavage [226]. Although no PCA ring cleavage pathways have been discovered in yeasts, the yeast *Arxula adeninivorans* converts PCA to catechol via a process catalyzed by Gdc [227]. It has been demonstrated that the yeasts *Candida parapsilosis* and *Trichosporon cutaneum* possess a second pathway in which PCA is converted to hydroxyquinol via the Mnx1 [228]. Figure 24 illustrates a schematic representation of the PCA and catechol degradation pathways in yeast. Tsai and Li [229] demonstrated that *Candida albicans* utilizes the catechol 1,2-dioxygenase (Hqd2) to cleave the aromatic ring of catechol. This enzyme is substrate specific and does not catalyze hydroxyquinol or catechol-related compounds. Hydroxyquinol is derived from a variety of aromatic compounds, including *p*-hydroxybenzoic and vanillic acids. It has been discovered that hydroxyquinol rings can be broken down in two distinct pathways. Maleylacetate is formed by oxidative cleavage of the intradiol ring. It is then converted to acetyl-CoA and succinate, which enter the TCA cycle via the first pathway [230]. The second pathway converts hydroxyquinol to 2,4-dihydroxymuconic semialdehyde, which is then converted to acetylpyruvate and formate via a series of reactions. Finally, acetylpyruvate is converted to acetate and pyruvate, which are then processed and utilized in the TCA cycle. The hydroxyquinol dioxygenase 1 (Hdx1) enzyme in the yeast *Candida parapsilosis* catalyzes the hydroxyquinol intradiol ring cleavage to form maleylacetate, which is further broken down into 3-oxoadipate and 3-oxoadipate-SCoA [228]. Gentisate dioxygenase (Gdx1) is a *Candida parapsilosis* yeast enzyme that catalyzes the cleavage of gentisic acid's aromatic ring to form maleylpyruvate [228]. The enzyme fumarylpyruvate dioxygenase 1 (Fph1) converts maleylpyruvate to fumarylpyruvate, which is then split into fumarate and pyruvate. It is worth noting that pyrogallol is converted to 2-hydroxymuconic acid in the yeast *Arxula adeninivorans* [227], and the pathway is identical to that described in detail for the catechol extradiol ring cleavage pathway.

In conclusion, the efficient wood-degrading process evolved in termite gut systems provides support to a novel concept that complete lignin degradation. Numerous species have been identified with aromatic metabolic pathways. However, one can speculate that the natural habitat of a microorganism has a significant effect on the metabolic pathways that are activated. The majority of aromatic metabolic enzymes in bacteria have been identified, whereas only a few have been identified in yeasts. In many microorganisms, the ferulic acid metabolic pathway is important because of the interaction of ferulic acid with polysaccharides such as xylan and pectin. This interaction could explain the overlap between the ferulic acid metabolic pathways of bacteria and fungi. However, it appears that fungi and yeasts have their own set of pathways that are distinct from others. The knowledge gained about wood-feeding termites could be used to improve and modify existing biomass pretreatment technologies. In addition, combining nature's and mankind's achievements should result in new scientific and technological breakthroughs that are critical for using aromatic wastes as renewable resources for producing biofuels and bioproducts.

## Some key scientific questions or challenges we have to face

The use of the non-natural yeast cells and their enzymatic/metabolic system for lipid synthesis is a clean, efficient, and renewable path to achieve "a sustainable development" towards biodiesel production. With this hypothesis, we have to face the following challenges in terms of science and the potential applications:

I. Lack of the potent/robust oleaginous yeast species or strains served as a potential cell factory to produce lipids from various aromatic waste material, such as azo dye, lignin analogous wastes, with this demand, we need to develop a technology system to screen and evaluate those potential robust oleaginous yeasts.
II. A complicated and unknown network regulation system commonly presented in the natural oleaginous yeast cells, with the demand to develop a robust oleaginous yeast cell factory for lipid conversion from waste biomass/organic dyes, we have to face the challenges for how to rebuild the yeast metabolic pathway with the synthetic biology methods in terms of the well understanding yeast regulation network by various "omics" technologies.
III. How to develop an optimized fermentation system of oleaginous yeasts to maximize the function of yeast lipid conversion capability from those aromatic biomass wastes is a fundamental challenge for us, where the yeast cell growth factors and some relevant co-factors in fermentation should be well understood and managed.

## The novel solutions and methodology we will propose

### Aromatic wastes can be efficiently utilized by oleaginous yeasts

To develop the biodiesel industry in a sustainable manner, and to truly industrialize it, we must address three issues comprehensively: the first is diversified raw material sources; the second is diversified conversion and utilization production technology; and the third is diversified product development. Oleaginous yeasts are capable of bio-converting lignin into lipids, the raw material used to make biodiesel. Cs-fad2, a Δ 12-fatty acid desaturase gene, was heterologously expressed in *Saccharomyces cerevisiae* for linoleic acid biosynthesis [231]. Increased inoculum size can increase *Cryptococcus curvatus*’ tolerance for phenolic compounds found in lignin and result in increased production of lipids. Vanillin was the most toxic, whereas *p*-hydroxybenzaldehyde and syringaldehyde inhibited *Cryptococcus curvatus* at concentrations ranging from 0–1.0 g/l [232]. Hu et al. [233] reported that *Trichosporon cutaneum* fermented on lignin model compounds (4-Hydroxybenzaldehyde) produced 0.85 g/l of lipids, with the major fatty acids being 42.9% stearic acid, 36.6% oleic acid, and 20.5% palmitic acid.

Numerous oleaginous yeasts, including *Cryptococcus curvatus*, *Rhodosporidium toruloides*, and *Yarrowia lipolytica*, have been found to grow on hydrolysates of inedible lignocellulosic biomass [234, 235]. When hydrolysates of the non-edible crops Cassava and *Jerusalem artichoke* were provided, *Rhodosporidium toruloides* could produce 63.2% and 56.5% lipid content (w/w), respectively [118]. In the United States, Asia, and Europe, the most promising non-edible lignocellulose biomasses/feedstocks are sugarcane bagasse, sugarcane husk, wheat straw, rice straw, and corn stover [236–241]. *Rhodotorula mucilaginosa* IIPL32 produced 15.3 g/l biomass and 0.17 g SCO per gram of xylose consumed when grown on the pentose fraction of acid pretreated sugarcane bagasse as a carbon source [242]. When *Rhodosporidium toruloides* was grown on a nonsterile distillery and domestic mixed wastewater medium, it produced 36.9% lipid content [243]. *Rhodosporidium toruloides* used acetic acid as another low-cost carbon source, accumulating 48.2% lipid content with 4.35 g/l cell dry biomass [244]. *Rhodotorula kratochvilovae* HIMPA1 is an oleaginous yeast with the unique ability to use pulp and paper industry effluent as a culture medium and accumulate a high amount of cell dry weight (13.87 g/l) with a total lipid yield of 8.56 g/l within the cellular compartment [243]. As a result, oleaginous yeast utilization has received a lot of attention.

### Termite guts as an unexplored reservoir of aromatics-degrading yeasts

Termites proliferate in large numbers in terrestrial ecosystems [22]. While the mechanisms of cellulose digestion in termites have become more understood, the degradation of lignin and aromatics remains a mystery. Termites play an important role in the global carbon cycle due to their ability to degrade lignocellulose; thus, lignocellulose-degrading enzymes from the "termite digestome" have many potential bioenergy applications [245, 246]. With the removal of cellulose (74–99%), hemicellulose (65–87%), and lignin (5.0–83%), termite digestion of lignocellulose is highly efficient, with typical bioconversion exceeding 95% within a day [24]. Recent research indicates that termite symbionts contain cellulolytic or lignin-derived components and are capable of degrading aromatic hydrocarbon compounds [247]. Thus, termite symbionts would be advantageous for industrial purposes. Despite the longer reaction time and more stringent control of microbial growth conditions, a biological system involving termite gut symbionts appears to be more appealing.

Several species of host organisms have been recently identified, including *Reticulitermes chinensis*, *Coptotermes formosanus*, and *Mastotermes darwiniensis*, while the newly isolated yeasts represent an untapped source of bioactive compounds, enzymes, and derived biotechnological applications. Intestinal yeasts can effectively remediate textile wastewater and decolorize both individual and mixtures of azo dyes, owing to their enzymatic activity and tolerance to high salt concentration [25, 26]. Concurrently, yeasts from wood-feeding termite guts constitute effective lignin, cellulose, and hemicellulose degraders and can be further exploited for the valorization of recalcitrant lignocellulosic biomass, as in the case of the xylanolytic *Candida pseudorhagii*, an efficient bioethanol producer upon xylose fermentation [96]. Furthermore, additional yeast benefits have been recently highlighted, including cold adaptation and tolerance to lignin-derived inhibitors [2, 27]. Lastly, novel yeast consortia can be efficiently constructed, with improved performance compared to the included individual isolates, mainly *Yarrowia* sp., *Meyerozyma guilliermondii*, *Sterigmatomyces halophilus*, *Barnettozyma californica*, *Meyerozyma caribbica*, *Debaryomyces hansenii*, and *Vanrija humicola*, or by combining the activity of yeast and bacterial isolates [96, 97].

## Reconstruction of oleaginous yeast metabolic network/pathway can be a powerful strategy to enhance lipid accumulation/transformation from aromatics

**Fig. 25:**
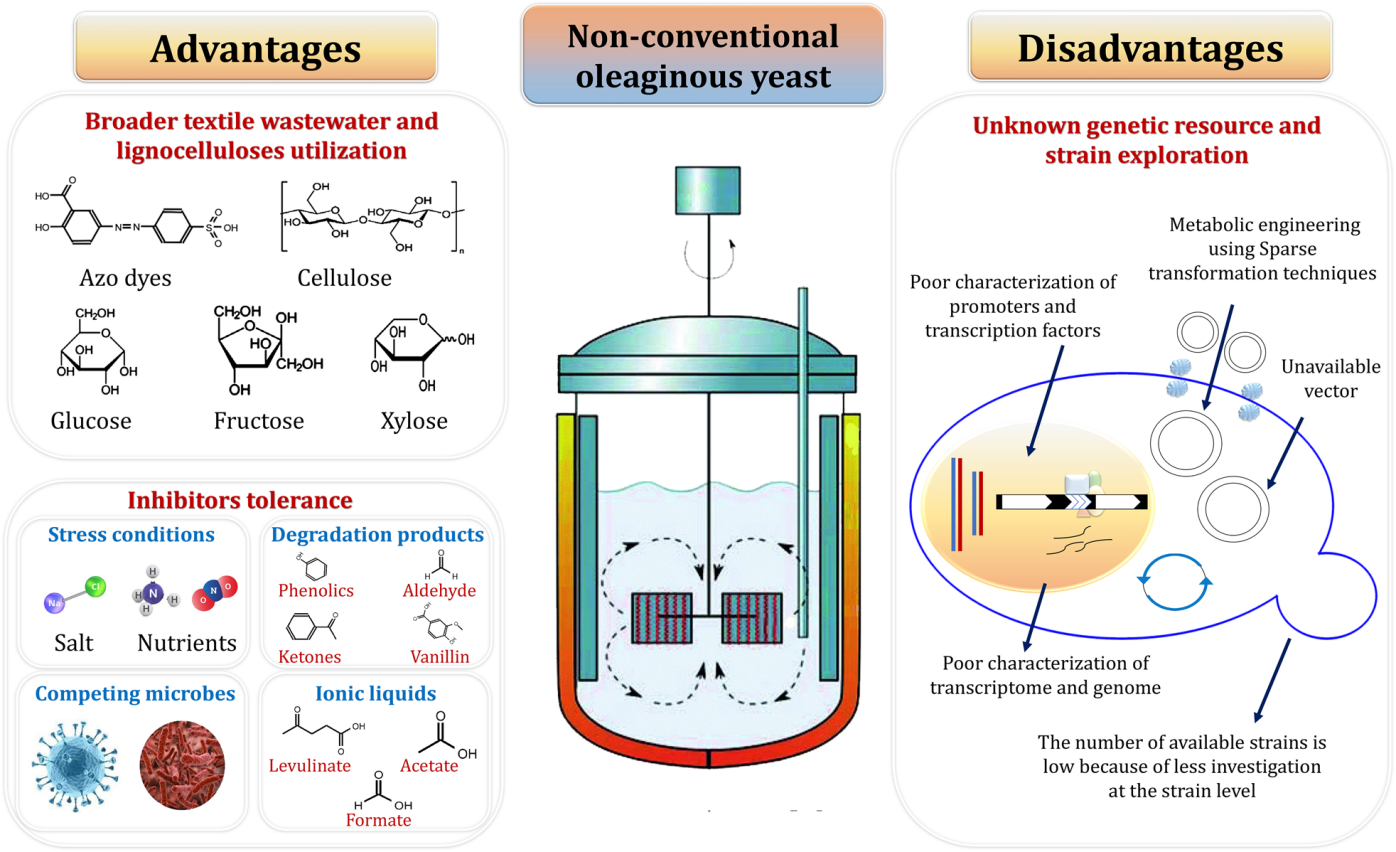
The advantages and disadvantages of using nontraditional oleaginous yeasts

The limitations of using oleaginous yeasts in biodiesel production include limited feedstock availability and falling short of theoretical lipid yields. These constraints could be overcome by screening and identifying potential alternative oleaginous yeast species, as well as investigating their industrial applications. The use of these alternative yeasts would theoretically improve hydrolysate specificity, carbohydrate utilization, and tolerance to osmotic pressures, pH changes, and growth inhibitors, lowering the cost of oleochemical production and, thus, end-product prices. Furthermore, by avoiding stringent pretreatment of feedstocks and working with strains that can tolerate competition from microbes in the surrounding environment and input waste, flexible culture conditions can be developed (Fig. 25). In recent years, based on omics and synthetic biology, the use of oleaginous yeasts to transform lignin biomass waste (and organic dye wastewater) into biodiesel products has become a new research hotspot and technological development direction, with good prospects for industrialization application [2, 18, 27, 28]. Preliminary research results have shown that high-oily yeast or a composite flora combination of different yeasts can not only efficiently degrade lignin waste and a variety of organic dye wastewater similar in structure to lignin, but also through biotransformation and fermentation. Treatment to achieve the purpose of decolorization and detoxification; at the same time, metabolites of lignin biomass or organic dyes can be effectively converted into high-quality biodiesel products in the metabolic process, kill two birds with one stone. Compared with the requirements of microalgae raw materials, the requirements of oleaginous yeast for raw materials do not have seasonality and higher energy and equipment input, and the economic performance of industrialization is higher.

**Fig. 26:**
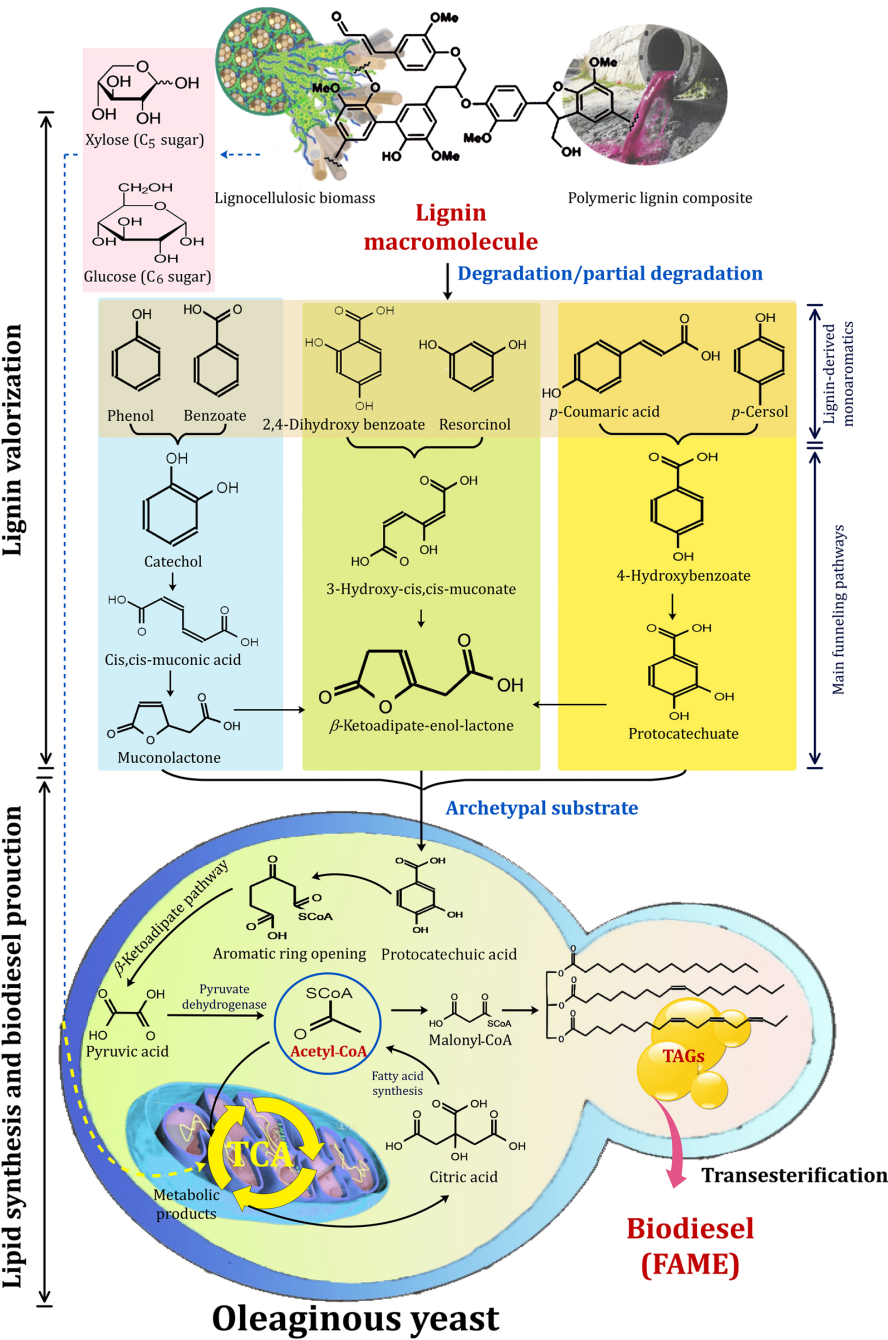
The proposed metabolic pathway for aromatics conversion into biodiesel by oleaginous yeast. Adapted from Ref. [18]

*Yarrowia lipolytica*, an oil-producing yeast, has attracted much attention due to its ability to accumulate high lipid content using a variety of substrates and its flexibility in genetic manipulation [248]. The intracellular lipid metabolism in *Yarrowia lipolytica* was customized to produce fatty acids, which are renewable lipid chemicals and precursors for the production of a new generation of biofuels [249]. The aromatic products of lignin depolymerization contain a lot of monocyclic and polycyclic aromatic compounds [250]. The synthesis of lipids in *Yarrowia lipolytica* was a complex metabolic network which is composed of lignin depolymerization pathways and fatty acid synthesis pathways [251]. Elucidating the metabolic network in *Yarrowia lipolytica* from lignin depolymerization to fatty acid synthesis is an important prerequisite for improving lignin conversion and constructing high-yield fatty acid-producing oleaginous yeast. Recently, we found that oleaginous yeast can effectively biodegrade lignin and its structural analogs of organic dyes and synthesize neutral oils efficiently. Figure 26 shows the proposed metabolic route of the lignin and lignin-like dye valorization into biodiesel by lipid-accumulating oleaginous yeast [18]. In addition, the physical and chemical quality of its synthetic biodiesel is also competitive (Table 2) [2]. The oleaginous yeast *Meyerozyma caribbica* SSA1654 was screened from wood‑feeding termite gut symbionts. Its synthetic oil conversion ability can reach up to 0.08 g/l/h, and the oil content of yeast can reach 47.25% (w/w) [2], showing attractive prospects for industrialization applications.

**Table 2:** Biodiesel properties produced by *Meyerozyma caribbica* SSA1654 in comparison with those of olive oil and the international biodiesel standard EN 14,214

Biodiesel properties	*M. caribbica* SSA1654	Olive oil	EN 14214
Long chain saturated factor (%, wt)	3.34	4.2	Not specified
Kinematic viscosity (mm^2^/s)	3.98	4.5	3.5–5.0
Iodine value (gI_2_/100 g)	92.37	84.0	≤ 120
Cetane number	52.32	57.0	≥ 51
Saponification value (mg KOH)	199.4	Not determined	Not specified
Linolenic acid (C18:3)	4.07	0.6	≤ 12
Degree of unsaturation (%, wt)	95.85	92.7	Not specified
Density (g/cm^3^)	0.88	Not specified	0.86–0.9
Oxidation stability (h)	8.3	3.3	≥ 6

Data obtained from Ali et al. [2]

## Technical bottlenecks/challenges and proposed solutions

**Fig. 27:**
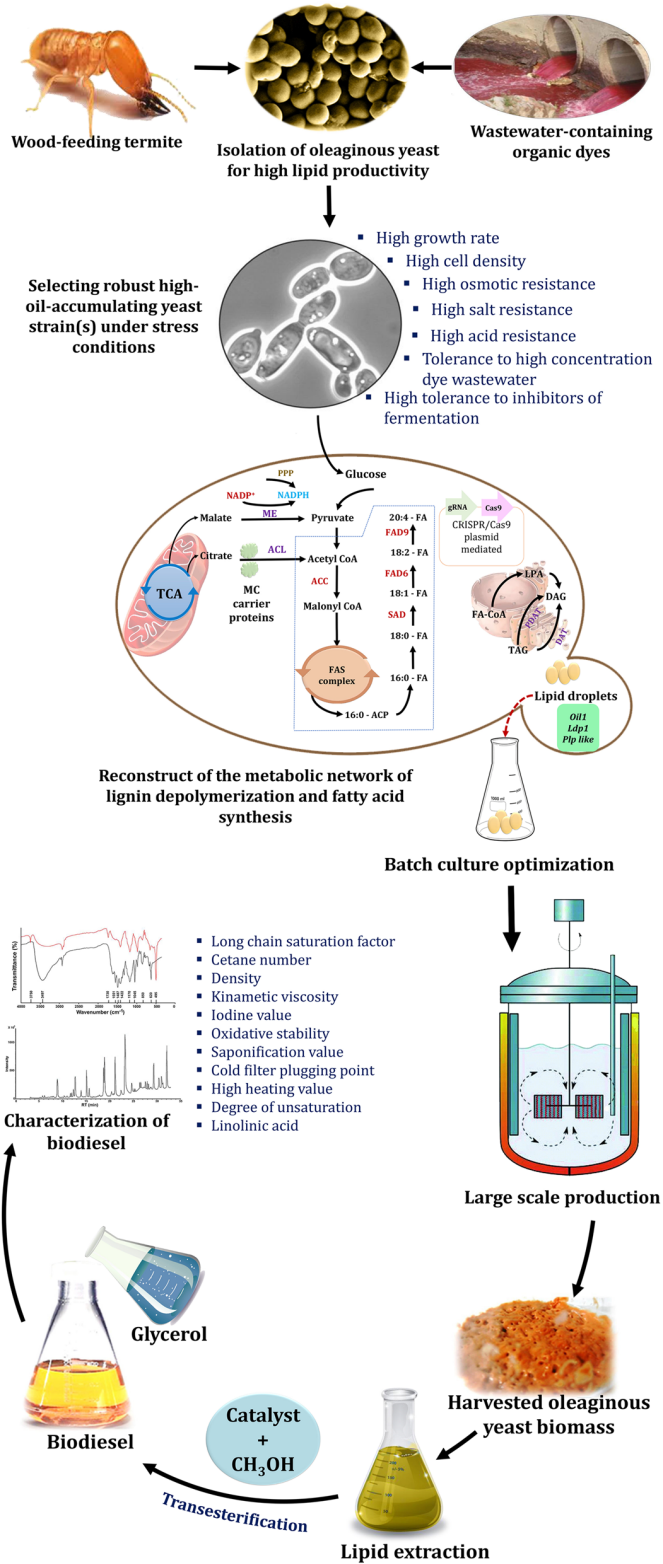
The proposed road map and methodology for producing biodiesel from aromatics-degrading oleaginous yeast inhabiting wood-feeding termites

Although yeast transforming aromatic wastes (lignin biomass and organic dye wastewater) into biodiesel has a certain potential for industrialization, there are still several technical bottlenecks and challenges that we need to face and solve through the following points. Figure 27 depicts a proposed road map and methodology for producing biodiesel from aromatics-degrading oleaginous yeast inhabiting wood-feeding termites.

## Strain screening and modification

The lipids produced from oleaginous yeast species have similar composition and energy values to plant and animal oils, thus, they may offer alternatives to several vegetable oil-based applications with no competition for food resources. They can become ideal bioeconomic feedstocks if grown using low-cost raw waste materials, such as lignin and its derivatives (e.g., lignocellulosic biomass and organic dye wastewater) and, thus have also a role in waste management. Therefore, the first proposed solution is how to construct a breeding technology and a comprehensive evaluation system for high lipid productivity-producing oleaginous yeasts. This challenge will be carried out by screening and identification of potential alternative oleaginous yeast species, as well as exploration of their industrial applications, may be required to overcome limitations, such as limited feedstock accessibility and falling short of theoretical lipid yields. Identification of robust oleaginous yeast strain(s) with specific characteristics, such as lignin and its aromatic-based derivatives utilization, tolerance to osmotic pressures, tolerance to pH changes, tolerance to growth inhibitors, and tolerance to high concentration dye wastewater, is therefore critical.

## A proposed novel metabolic pathway and strategy

Lipid metabolism has evolved in eukaryotes as a complex metabolic network with a large number of enzyme-catalyzed reactions connecting different compartments of the cell. In terms of fatty acid precursors and corresponding enzymes, there are mechanistic differences between oleaginous and non-oleaginous yeast species, which are tightly linked with other metabolic pathways and regulated at various branch points. As a result, the enzymes involved in lipid biosynthesis are important targets for increasing lipid accumulation. A few oleaginous yeast species, such as *Yarrowia lipolytica*, *Rhodotorula toruloides*, and *Lipomyces starkeyi*, have recently gained popularity and are now used for a variety of industrial applications. The availability of abundant genetic resources, optimized culture conditions, and insights into physiological, biochemical, and molecular responses to varying growth conditions have aided in the development of tailored gene-editing technologies and amenable transformation methods for these species. Despite extensive research, they have some drawbacks, particularly access to limited feedstock and falling short of theoretical lipid yields. Therefore, the second proposed solution is how to reconstruct the metabolic pathway/network of the process of yeast transforming aromatic wastes to maximize the lipid transformation and accumulation rate from the lignin and lignin-based aromatics with the applications of various “omics” technologies or a synthetic biology approach. This challenge will be carried out on selected oil-producing yeast strain as below.

## Multi-omics analysis

To elucidate the metabolic network of aromatics depolymerization and fatty acid synthesis, the combined analysis of transcriptomics, proteomics and metabolomics will be performed. Through omics analysis, the key pathways, catalytic enzymes, regulatory factors and key intermediate metabolites involved in the degradation of aromatic compounds and fatty acid synthesis will be identified. Based on the combined omics analysis, the metabolic network from aromatics to fatty acid in oleaginous yeast will be elucidated.

## The research for the stress response mechanism of oleaginous yeast in the presence of aromatics

When aromatic is used as a substrate, the stress response mechanism of oleaginous yeast to environmental stress is analyzed by comparative multi-omics analysis. Due to the cytotoxicity of lignin and its derived aromatic compounds, it is necessary to clarify the variation of gene expression profile, transcription levels of key enzymes and transcription regulators, expression levels of enzymes in the presence of these aromatic compounds, and the variation of the transmembrane transport system in response to the environmental stress. This proposed solution will help to understand the physiological characteristics of yeast cells in the presence of lignin and its derived aromatic compounds and provide a theoretical basis for the genetic engineering of yeast cells.

## Construction of high fatty acid-producing strain

In order to improve the efficiency and accumulation of fatty acid synthesis, metabolic network reconstruction, including overexpression of genes, will be performed to improve the precursor supply of fatty acid biosynthesis, reduce the consumption of fatty acid in natural pathway of robust yeast, and promote the excessive synthesis of fatty acid through: (i) increasing the synthesis of acetyl-CoA; (ii) deleting the genes responsible for fatty acid activation and peroxisome transport via gene knockout; and (iii) overexpression of target genes to increase fatty acid production.

## Conclusion and perspectives

### Innovation points and future technology development

I. This review proposed a novel concept in utilization oleaginous yeasts as the cell factory to convert aromatic wastes to lipid, indicating a completely new type of feedstock potentially valuable for commercial biodiesel product using the robust yeasts explored in the gut of wood-feeding termites.
II. This review also proposed a novel strategy for rebuilding the metabolic pathway of oleaginous yeasts in converting aromatic wastes to lipids, with the goals of (1) analyzing the genome characteristics of the targeted yeast; (2) developing a new base mutation gene editing technology; and (3) clarifying the impact of the insertion position of aromatic compounds and other biosynthetic pathways in the industrial chassis genome on the expression level and genome stability.
III. This review has uniquely designed a new strategy to adopt advanced "multi-omics" technology for a better understanding of the metabolic regulation mechanism of those targeted yeasts regarding its regulation processing in depolymerizing lignin and other aromatic textile dyes, where it will further allow us to easily identify a variety of involved enzyme functions, and further realize the reconstruction of the metabolic network of the targeted compounds, and eventually improve the ability of oil synthesis and transformation.
IV. This review developed and evaluated a new set of standards and approaches for screening and evaluating robust oleaginous yeasts, as well as the quality of terminal biodiesel product converted from aromatic wastes.

### Values in science and industry

Aromatics, which include lignin biomass and azo dye wastewater, are the most abundant renewable aromatic resources in nature, but they are also a source of organic pollutants. Three major issues for Egypt/China are how to adequately utilize such abundant aromatics and environmental pollutants to obtain a satisfying production capacity, how to reasonably use such biodiesel in each region, and how to scientifically encourage the development of Egyptian/Chinese biodiesel industries. *Meyerozyma caribbica*, *Yarrowia lipolytica*, and other oil-producing yeast strains we have isolated from termite gut symbionts, in recent, has received a lot of our attention due to its ability to accumulate high lipid content using a variety of substrates. Therefore, developing a novel bioconversion pathway from lignin and environmental pollutants to biodiesel using robust aromatics-degrading oleaginous yeasts as a cell factory is a promising exploration in terms of its scientific and industrial values.

## Data Availability

Not applicable.

## Abbreviations

CO_2_: Arbon dioxide
TAG: Triacylglycerides
AAs: Aromatic amines
CO: Carbon monoxide
HC: Hydrocarbons
PM: Particulate matter
SO_2_: Sulphur dioxide
NOx: Nitrogen oxides
GHG: Greenhouse gases
WOCs: Waste cooking oils
LUC: Land use change
SCO: Single cell oils
VFAs: Volatile fatty acids
-N = N-: Azo bond
PAHs: Polycyclic aromatic hydrocarbons
COD: Chemical oxygen demand
BOD: Biological oxygen demand
PCA: Protocatechuic acid
Hqd2: Catechol 1,2-dioxygenase
Hdx1: Hydroxyquinol dioxygenase 1
Gdx1: Gentisate dioxygenase
Fph1: Fumarylpyruvate dioxygenase 1
PDC1: Pyruvate decarboxylase
R5P: Ribulose-5-phosphate
SCT1: Glycerol-3-phosphate acyltransferase
OAA: Oxaloacetate
TAL: Transaldolase
LRO1: Phospholipid/diacylglycerol acyltransferase
SLC1: Lyso-phosphatidic acid acyltransferase
RPE: Ribulose 5-phosphate 3-epimerase
ACAD: Acyl-CoA dehydrogenase
3-HCAD: 3-Hydroxybutyryl-CoA dehydrogenase
TKL: Transketolase
ECAH: Enoyl-CoA hydratase
LPA: Lysophosphatidate
FBPA: Fructose-bisphosphate aldolase
XR: Xylose reductase
FBP: Fructose 1,6-bisphosphate
PK: Phosphoketolase
GND2: 6-Phosphogluconate dehydrogenase
IDH: Isocitrate dehydrogenase
AKG: α-Ketoglutarate
ENO: Enolase
PGI: Phosphoglucose isomerase
PGK: Phosphoglycerate kinase
PGL: 6-Phosphogluconolactone
PTA: Phosphotransacetylase
XL: Xylonolactonase
3PG: 3-Phosphoglycerate
G3P: Glyceraldehyde-3-phosphate
G3PD: Glyceraldehyde-3-phosphate dehydrogenase
G6P: Glucose-6-phosphate
SOL3: 6-Phosphogluconolactonase
PA: Phosphatidate
ACC1: Acetyl-CoA carboxylase
ACL1/ACL2: ATP-citrate lyase
LsFad2/LsFad3: Fatty acid desaturase
2PG: 2-Phosphoglycerate
RPI: Ribose-5-phosphate isomerase
FAS1/FAS2: Fatty acid synthase subunit
MDH1: Malate dehydrogenase 1
PL: Phospholipids
ALE1: Acyltransferase for lysophosphatidylethanolamine
XD: Xylonite dehydratase
GPD1: Gly3P dehydrogenase
GUT2: Gly3P dehydrogenase
ACS: Acetyl-CoA synthetase
XI: Xylose isomerase
PGM: Phosphoglycerate mutase
PFK: Phosphofructokinase
DGA1/DGA2: Diacylglycerol acyltransferase
PAH1: Phosphatidate phosphatase
LsElo1/LsElo2: Fatty acid elongase
AADH: Acetylating acetaldehyde dehydrogenase
F6P: Fructose-6-phosphate
HXK: Hexokinase
PYK: Pyruvate kinase
ALD: Acetaldehyde dehydrogenase
ADH: Alcohol dehydrogenase
MAE1: Malic enzyme
DAG: Diacylglycerol
DGK1: Diacylglycerol kinase
PG: 6-Phosphogluconate
XDH: Xylitol dehydrogenase
PP: Phosphoenol pyruvate
XK: Xylulokinase
ADH: Alcohol dehydrogenase
